# Stoichiometry-controlled secondary structure transition of amyloid-derived supramolecular dipeptide co-assemblies

**DOI:** 10.1038/s42004-019-0170-z

**Published:** 2019-06-13

**Authors:** Wei Ji, Chengqian Yuan, Priyadarshi Chakraborty, Sharon Gilead, Xuehai Yan, Ehud Gazit

**Affiliations:** 1 George S. Wise Faculty of Life Sciences, Department of Molecular Microbiology and Biotechnology, Tel Aviv University, Tel Aviv 6997801, Israel; 2 State Key Laboratory of Biochemical Engineering, Institute of Process Engineering, Chinese Academy of Sciences, Beijing 100190, China; 3 Iby and Aladar Fleischman Faculty of Engineering, Department of Materials Science and Engineering, Tel Aviv University, Tel Aviv 6997801, Israel

## Abstract

Conformational transitions of secondary structures are a crucial factor in many protein misfolding diseases. However, the actual transition of folded proteins into β-sheet-rich structures is not fully understood. Inhibition of aggregate formation, mediated by the β-sheet conformation, and control of the secondary structural transition of proteins and peptides could potentially attenuate the development of amyloid-associated diseases. Here we describe a stoichiometry-controlled secondary structure transition of amyloid-derived dipeptide assemblies from a β-sheet to supramolecular helix conformation through coassembly with a bipyridine derivative. The transition is mainly mediated by the intermolecular hydrogen bonds and π-π interactions between the two components, which induce the altered stacking and conformation of the co-assemblies, as confirmed by experimental results and computational simulations. This work not only exemplifies a feasible strategy to disrupt the β-sheet conformation, underlying amyloid-like fibril formation, but also provides a conceptual basis for the future utilization of the helical nanostructures in various biological applications.

β-sheets and helices (e.g. α-helix and supramolecular helix) are the major secondary structure motifs that facilitate the organization of the three-dimensional conformation of proteins and peptides^1^. Supramolecular assemblies comprising β-sheet-rich fibril structures are associated with various amyloid degenerative disorders, such as Parkinson’s disease, Alzheimer’s disease, and type 2 diabetes^2–6^. Both random coil to β-sheet and helix to β-sheet transitions could occur during aberrant protein folding and assembly, resulting in deposition of insoluble protein fibrils, leading to cell and organ dysfunction, and in many cases, to apoptosis^7–13^. However, understanding of the structural behavior of β-sheet assemblies in amyloid fibrils remains poorly understood. On the other hand, supramolecualr helix with inter- and intra-hydrogen bonding is crucial for structural support, playing a role in mechano-transduction and cell motility, and in defining the cell’s stretchiness^14–16^. Thus, the transformation of β-sheet to helix not only bears important implications for disrupting and reversing amyloid formation, but may also lay the basis for the use of supramolecular helix structures in various applications, such as DNA binding, membrane spanning, tissue engineering, etc^17^.

To fully understand the conformational transitions of proteins and peptides (e.g. the transition between β-sheet and helix), several strategies have been reported aiming to control the structural conversion and shift the equilibrium in either direction by changing various conditions^18^, including temperature^19,20^, pH^21^, ion strength^22^, the presence of metal ions^23,24^, X–N Acyl migration^25^, and co-assembly with additives^26,27^. The coassembly approach is particularly attractive since supramole-cular non-covalent interactions, such as hydrogen bonding, π–π stacking, van der Waals interactions, and hydrophobic interactions^28–41^, are usually influenced and determined by the stoichiometric ratio of the system components, leading to completely different stacking and switched structural conformations. However, analysis of secondary structure transition of amyloid-like fibrils from β-sheet to helix triggered by the co-assembly approach remains extremely challenging.

Taking a reductionist approach, short peptide fragment models have provided novel insights into the study of amyloid formation^42,43^. Specifically, Fmoc-protected diphenylalanine (FmocFF), containing the FF central recognition motif of the Alzheimer’s β-amyloid polypeptide, self-assembles into ordered and discrete antiparallel β-sheet rich nanofibrils through strong aromatic-aromatic interactions and hydrogen bonding^44–47^. Previous studies have reported that FmocFF nanofibrils show amyloid-like structural signatures, such as characteristic morphology, secondary structure conformation, and Congo red binding^48,49^. Based on the strong hydrogen bonding between carboxylic acid and pyridine^50,51^, FmocFF self-assembly may be easily modulated by intermolecular hydrogen bonds between two co-assembled components, thereby inducing secondary structural transformation through co-assembly with pyridine derivatives.

Here we present a simple approach to control the secondary structure transition of amyloid-derived FmocFF assemblies from a β-sheet into a helix structure through stoichiometric coassembly with 4,4-bipyridine (BPY) (Fig. 1). The secondary structures and aggregation states of the co-assemblies are investigated by circular dichroism (CD), transmission electron microscopy (TEM) and Fourier transform infrared (FTIR). β-sheet to helix transition of the co-assemblies is obtained by modifying the molar ratios of FmocFF/BPY from 1:1 to 2:1. The formation of intermolecular hydrogen bonds between carboxylic acid in FmocFF and pyridine in BPY is mediated by the initial ratios of the two molecules, which determine the threedimensional arrangement and influence the structural conformation. The packing and dynamic secondary structure formations are simulated by all-atom molecular dynamics (AAMD) simulations for both β-sheet and helix co-assemblies. This work exemplifies a feasible method to inhibit the formation of β-sheet structures comprising amyloid-like fibrils through a coassembly approach and may lay the basis for future biological applications of helical nanostructures.

## Results

### Co-assembled supramolecular hydrogels

Owing to the number of carboxylic acid group and pyridine in FmocFF and BPY, we believe that two molar ratios (1:1 and 2:1) for FmocFF/BPY will be ideal for intermolecular hydrogen bonding interactions. Both systems (FmocFF/BPY, 1:1 and 2:1) formed self-supported and invertible gels, similar to single-component FmocFF system, at combined concentrations of 2.0 mg/mL in H_2_O/DMSO (v/v = 98:2), while single component BPY was completely dissolved in this solution (Supplementary Fig. 1). We further recorded the details of optical images of FmocFF and FmocFF/BPY (1:1 and 2:1) gels to study the dynamic behaviors of co-assemblies over time, as well as the formed nanostructures, using TEM. In the single-component gel, emulsion of FmocFF was observed at zero timepoint and becomes clear gradually within 10 min upon completion of the self-assembly process, producing an optically transparent gel (Supplementary Fig. 2). However, the relatively opaque 1:1 and 2:1 FmocFF/BPY, the solutions were converted to completely transparent gels after 32 and 45 min, respectively. The single component BPY solution was clear throughout the experiment. These results suggest co-assembly between FmocFF and BPY in the two-component gels as well as different coassembly kinetics for hydrogels with different molar ratio of FmocFF/BPY (1:1 and 2:1).

**Fig. 2:**
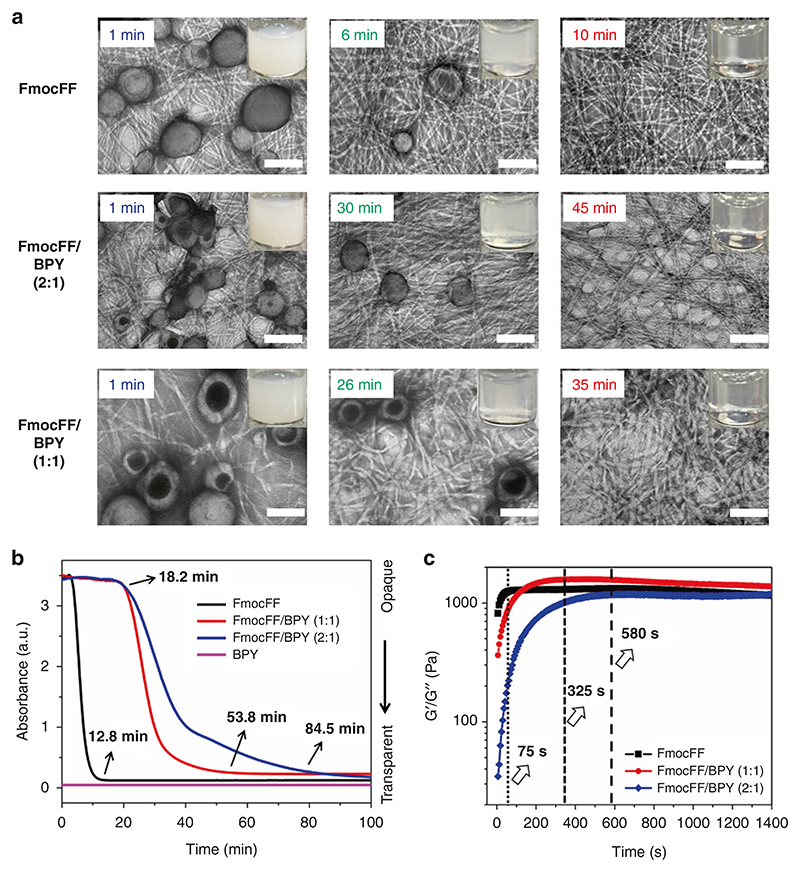
Co-assembly of FmocFF with BPY. **a** Time lapse optical images and the corresponding TEM images of FmocFF, FmocFF/BPY (2:1,1:1) in H_2_O/DMSO (v/v = 98:2) at a concentration of 2 mg/mL for different time points. Scale bar is 250 nm. **b** Turbidity measured at 405 nm over 100 min for single FmocFF, BPY and FmocFF/BPY (2:1,1:1) solutions in H_2_O/DMSO (v/v = 98:2) at a concentration of 2 mg/mL. **c** Time dependent evolution of G’ values of the single FmocFF and FmocFF/BPY (1:1, 2:1) gels at constant frequency of 1 Hz and 0.1% strain

The micro-structures of FmocFF and FmocFF/BPY (1:1 and 2:1) were examined by TEM at three time points, namely timepoint zero, during gel formation and at the final gel stage (Fig. 2a). Addition of a transparent solution of Fmoc-FF in DMSO to water resulted in immediate turbidity of the mixture, which is due to the formation of large sizes of spherulite structures that are able to scatter light^52^. The spherulitic structures with the size of 100–300 nm acted as nucleation points and transformed to a uniform entangled fibrous network after ~10 min for FmocFF as observed from the TEM images. However, in the co-assembled gels, the sphere shape turned into well-defined fibers which entangled with each other for gel formation after 32 and 45 min for 1:1 and 2:1 FmocFF/BPY, respectively.

To quantify the kinetics of the assembly process, we monitored time dependent absorbance of these self- or co-assembly solutions at the wavelength of 405 nm (Fig. 2b). The absorbance of FmocFF solution starts to decrease after 2.7 min and reaches an optical density (OD) of 0.12 at the time point of 12.8 min, while the absorbance of the BPY solution remains around zero all the time. However, the absorbance of the co-assembled solution remained high up to 18.2 min, then gradually decreased until reaching an OD of around 0.22 after 53.8 and 84.5 min for FmocFF and FmocFF/BPY (1:1 and 2:1), respectively. The kinetics of fiber formation by the FmocFF/BPY solution is slower than that of FmocFF, proves that co-assembly indeed occurred in the two component systems, possibly regulated by the ratio of FmocFF/ BPY. It should be noted that there is a difference between the time frames of the vial turbidity assay and the absorbance measurement at 405 nm, which are possibly due to the different solution volumes used in each experiment affecting the assembly kinetics.

### Mechanical properties of hydrogels

In order to analyze the change in mechanical properties of the FmocFF gels after co-assembly with BPY, we studied their rheological properties. Strain sweep experiment was first carried out on the FmocFF, FmocFF/BPY (1:1 and 2:1) gels (Supplementary Fig. 3) to examine the linear viscoelastic region. It was evident that the co-assembled gels exhibited breakage (cross over of the storage (G’) and loss (G”) modulus values) at higher strain values compared to the FmocFF gel. Dynamic frequency sweep experiments (Supplementary Fig. 3) revealed that for all gels, the G’ and G” values were independent of the frequency, thus establishing their gel-like behavior. The co-assembled gels, although with a lower modulus, showed breakage at much higher strain values, as the interconnection zones between the fibers became highly flexible^53^. This may attribute to enhanced hydrogen bonding between BPY and FmocFF in the co-assembled fibrous gels. Next, we tried to evaluate the kinetics of gelation by time sweep experiments (Fig. 2c). Completion of the gelation procedure was assumed when the G’ values reached the plateau region. Gelation time increased in the 1:1 and 2:1 FmocFF/BPY gels (325 and 580 s, respectively) indicating slower kinetics, which are in good agreement with the results of transparency change of gels. Finally, we analyzed the recovery of mechanical properties of the gels after shear deformation by step strain experiments (Supplementary Fig. 3). All the gels exhibited sheer recovery properties, which were further verified over multiple cycles. Overall, the rheological data indicates co-assembly of FmocFF and BPY, conferring a significant effect on the mechanical properties of the gels, including their gelation kinetics. However, the co-assembly does not disturb the sheer recovery property of the FmocFF gels.

### Secondary structure and morphology of co-assemblies

**Fig. 3:**
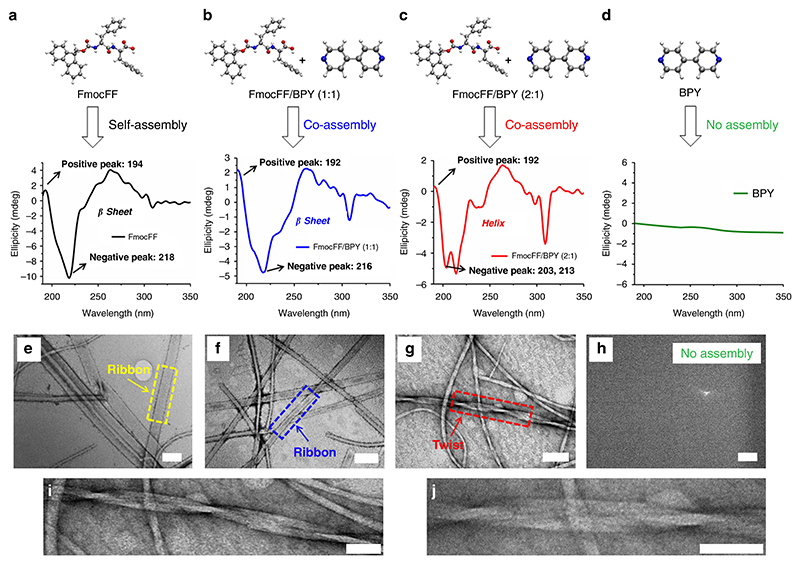
Secondary structures of FmocFF/BPY at different ratio conditions. (**a**–**d**) CD spectra and (**e**–**h**) high resolution TEM images. **a**, **e** FmocFF, **b**, **f** FmocFF/ BPY (1:1), and **c**, **g** FmocFF/BPY (2:1), **d**, **h** BPY. Scale bar is 100 nm. **i**, **j** High-magnification TEM images of helical twist fibril in **g**. Scale bar is 50 nm

To further study the secondary structures of the final assemblies, CD spectroscopy was employed. For the FmocFF hydrogel (Fig. 3a), positive and negative cotton effects were observed at 194 and 218 nm, indicating a β-sheets rich structure. After co-assembly with BPY at a 1:1 ratio (Fig. 3b), similar pattern and cotton effect bands were obtained at 192 and 216 nm, demonstrating a slight blue shift (2 nm), but also indicating a similarly β-sheet rich coassembly. Surprisingly, one positive peak at 192 nm and two negative peaks at 203 and 213 nm were observed by changing the ratio of FmocFF/BPY to 2:1 (Fig. 3c), suggesting a transition from β-sheet to helix conformation in the two-component hydrogel. Surprisingly, one positive peak at 192 nm and two negative peaks at 203 and 213 nm were observed by changing the ratio of FmocFF/BPY to 2:1 (Fig. 3c), suggesting a transition from β-sheet to helix conformation in the two-component hydrogel. The CD spectrum for FmocFF/BPY (2:1) shows a blue-shifted α-helix-like pattern in its minima, compared to longer α-helical peptides (222–213 nm, 208–203 nm), which has been observed before in short peptide helices. Baldwin and colleagues have shown that the negative maxima (208, 222 nm) shift to (205, 219), (204, 217) and (200, 215 nm) as the sequence becomes shorter from 11 to 8 to 4 helical peptide units, respectively^54^. In another study, Fairlie and colleagues showed that the CD signals of single turn α-helices usually appears at negative maxima of 207 and 215 nm^55^. Our group also reported a self-assembling single heptad repeat module to form a helical conformation, where the peptide exhibited two negative maxima at around 205 and 218 nm^56^. Previous studies have also explored the relationship between CD and the degree of twisting of β-sheets, showing that strongly twisted β-sheets generate a stronger red shift at the minimum and maximum wavelengths, but do not change the original nature of CD pattern, with one minimum negative band and one maximum positive band^57–59^.

Thus, the two blue-shifted negative maxima at 203 and 213 nm, along with the positive maximum at 192 nm, observed for FmocFF/BPY (2:1) should be ascribed to the helical conformation, rather than to a twisted β-sheet conformation. In contrast, single BPY showed an almost undetectable CD signal, because of the achiral property of BPY (Fig. 2d). A difference in peak intensity at 240 and 308 nm was also observed for the FmocFF/ BPY (2:1) gel, since peptide nanofibrous hydrogels are supra-molecular aggregates, and in some cases, they have different transparency, which is likely to result in some fraction of the light being scattered, thereby influencing peak intensity^58,59^.

High resolution TEM images of the FmocFF and FmocFF/ BPY (1:1) gels showed ribbon-like fibrils, 11–65 and 21–70 nm in width, respectively (Fig. 3e, f). However, helical twist fibrils 10–45 nm in diameter with a pitch of 88–110 nm were observed in the 2:1 FmocFF/BPY gel (Fig. 3g), induced by the completely different molecular packing. High-magnification of images showing the helical twist for 2:1 FmocFF/BPY gel was also depicted in Fig. 3i, 3j. No assembly was found for single BPY solution (Fig. 3h). To gain more insight into the effect of two components ratio on the secondary structures and morphologies, the 1.5:1 and 1:2 FmocFF/BPY gels were further studied by optical images and TEM images, showing solid fibrous gel, and fibrous half-gel, respectively (Supplementary Fig. 4). The CD spectrum of the 1.5:1 FmocFF/BPY gel showed two positive peaks at 193 and 225 nm and two negative peaks at 204 and 241 nm (Supplementary Fig. 4). One positive peak at 196 nm and one negative peak at 226 nm were observed for FmocFF/BPY (1:2) with red shifted CD signals (Supplementary Fig. 4), indicating more twisted β-sheet arrangements. Remaining BPY may form π-π interactions with fluorenyl or pyridine rings of dimer of 1:1 FmocFF/BPY and further increase the hydrogenbonding distance between two dimers, weakening the inter-molecular forces and hydrogen bonds on the periphery of the β-sheet^58,59^.

### Quantifications of secondary structures of co-assembly

**Fig. 4:**
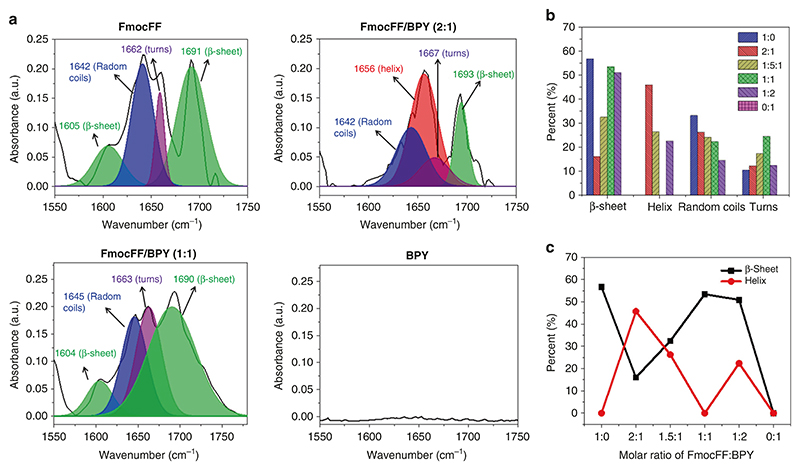
Quantifications of secondary structures by FTIR. **a** FTIR analysis of the amide I region, ranging from 1600 to 1700 cm-1, of FmocFF, BPY, and FmocFF/BPY mixtures (2:1,1:1) fit by multiple Gaussian peaks. **b** The secondary structural components and their proportion at different FmocFF/BPY ratios (1:0, 2:1, 1.5:1, 1:1, 1:2, 0:1). **c** Variation curves of the percent of β-sheet, anti-β-sheet, and helix pattern obtained by changing the ratio of FmocFF/BPY (1:0, 2:1, 1.5:1, 1:1, 1:2, 0:1)

To further explore the underlying mechanism and stoichiometry of FmocFF and BPY co-assembly, the FTIR spectra of self- or coassemblies at different molar ratios (single FmocFF, single BPY, and FmocFF/BPY = 2:1, 1.5:1, 1:1, 1:2) were examined in detail. Specifically, the stretching mode of C=O groups (amide I region) in the peptide backbone was analyzed to identify different types of secondary structures using solid-state FTIR spectroscopy^60–62^. FmocFF nanofibrils are a type of amyloid-like assembly and show four peaks in the amide I band, which can be ascribed to β-sheets (1605, 1692 cm^−1^), random coils (1642 cm^−1^), and turn structures (1662 cm^−1^). We found the assemblies of the FmocFF-based dipeptides system yield amide I bands at β-sheets (1610–1640 and 1685–1700 cm^−1^), whereas the helix conformation signals at 1650–1660 cm^−1^, and random coils and other turn structures show bands at 1640–1650, 1660–1685 cm^−1^, respectively^63^. Peak separation calculations were carried out through peak fitting of the amide I region ranging from 1600 to 1700 cm^−1^ and the proportion of each estimated secondary structure constituent is summarized in Fig. 4, Supplementary Fig. 5 and Supplementary Table 1. For the 1:0 single FmocFF or 1:1 FmocFF/BPY ratio, over 50% of β-sheet conformation (parallel β-sheet at ~1605 cm^−1^ and anti-parallel β-sheet at ~1690 cm^−1^) was observed, with no obvious helix. Since more twisted β-sheets were formed in the 1:2 FmocFF/BPY, as indicated by the by CD analysis, the peak of twisted β-sheet was assigned to 1650 cm^−159^. The total β-sheet conformation for 1:2 FmocFF/BPY was approximately 50%. However, some transition from anti-parallel β-sheet to helix (~26%) was observed by changing the FmocFF/BPY ratio to 1.5:1.

No secondary structure was found in the 0:1 single BPY due to the good solubility of BPY, showing no assembly in water. Interestingly, the highest proportion of helix (45.71%) was observed in the 2:1 FmocFF/BPY ratio, presumably due to the completely different stacking formed by co-assembly. These results exhibited that the secondary structures of β-sheet rich dipeptide assembly were regulated by tuning the ratio of FmocFF/ BPY and the highest transition of secondary structures from β-sheet to helix was observed at the condition of 2:1 FmocFF/BPY, which is in good agreement with CD spectra.

### Hydrogen bonding between the carboxylic group of phenylalanine and bipyridine

**Fig. 5:**
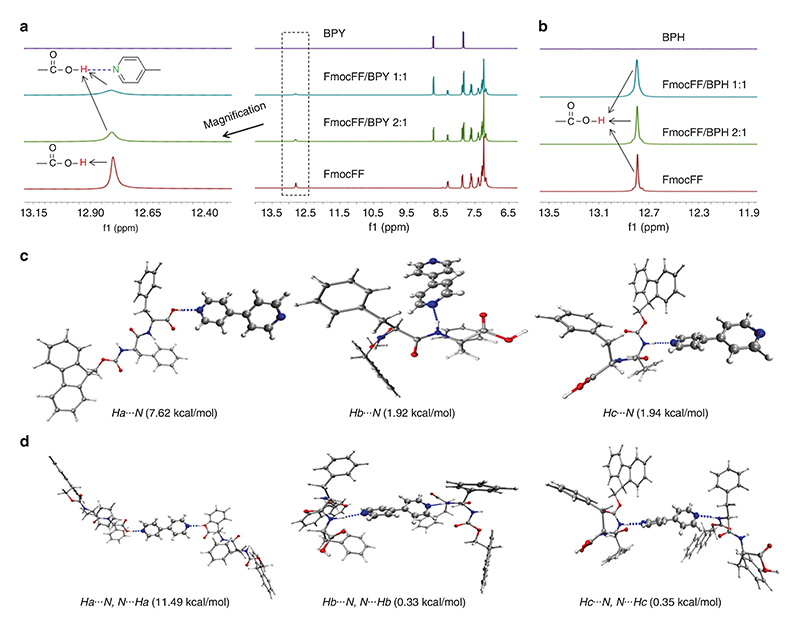
Hydrogen bonding between pyridine and carboxylic acid. **a** ^1^H NMR spectra of FmocFF and BPY with different equivalents of BPY in DMSO-*d*_6_. **b** ^1^H NMR spectra of FmocFF and BPH with different equivalents of BPH in DMSO-*d*_6_. **c**, **d** Multiple dimers and trimers of FmocFF and BPY connected by different hydrogen bonding interaction modes at a ratio of (**c**) 1:1 FmocFF/BPY and (**d**) 2:1 FmocFF/BPY. The corresponding binding energy is shown below the molecular packing patterns

Feng et al. reported the formation of strong hydrogen bonding between phenylalanine derivative (LPF) and bypridine (BPY) for regulating the supramolecular chirality of nanofibrils through co-assembly^50^. The co-crystal structure of LPF/BPY showed that one-dimensional polymer chain could be formed by the hydrogen bonding between the carboxylic group and pyridine, which further produce three-dimensional crosslinked networks through π-π interactions (Supplementary Fig. 6). For the FmocFF/BPY system, ^1^H NMR was employed to explore the hydrogen bonding interaction between the carboxylic acid and the pyridine of FmocFF and BPY, respectively. It should be noted that the pKa values of FmocFF and BPY are 3.50 and 3.27, respectively, and the ΔpKa [pKa (BPY)-pKa (FmocFF)] is -0.23 (<3.75) resulting in no proton transfer^64^. As shown in Fig. 5a, the proton signal assigned to the carboxylic acid group of FmocFF was broadened and greatly weakened upon adding BPY, demonstrating the strong hydrogen bonding interaction^51^. Compared to 2:1 FmocFF/BPY, a slightly broader and weaker peak of the acid proton was observed for 1:1 FmocFF/BPY, possibly due to the a slightly stronger hydrogen bonding, as indicated by density functional theory (DFT) calculations (Supplementary Fig. 7). Additionally, we also performed a control experiment of FmocFF/biphenyl (BPH) to study the behavior of the proton signal of carboxylic acid group without pyridine part (Supplementary Fig. 9). We observed only little change for the proton signal of carboxylic acid group in FmocFF after adding BPH (Fig. 5b). Temperature-dependent ^1^H NMR, studied to investigate the dynamic behavior of hydrogen adjacent to the nitrogen in BPY (Supplementary Fig. 10), exhibited an upfield shift due to the disassembly of hydrogen bonding between –COOH and BPY with increase in temperature in aqueous solution. The reversible property of this hydrogen bond triggered by temperature was also demonstrated by heating and cooling cycle. Moreover, DFT calculations were conducted to investigate the hydrogen bonding interactions between FmocFF and BPY. There are three types of hydrogen atoms (Ha, Hb, Hc) in the chemical structure of FmocFF, which can potentially form hydrogen bond with BPY (Supplementary Fig. 11). Multiple dimers and trimers of FmocFF and BPY (1:1, 2:1) connected by different hydrogen bonding interaction modes were depicted in Fig. 5c, d and Supplementary Fig. 8. At the condition of 1:1 FmocFF/BPY, it is evident that the O–Ha⋯N hydrogen bond possesses the shortest length, highest bond strength and binding energy (1.69 Å, 12.62 and 7.62 kcal/mol) compared to both N-Hb’⋯N (2.03 Å, 5.27 and 1.92 kcal/mol) and N–Hc⋯N (2.03 Å, 5.24 and 1.94 kcal/mol) hydrogen bonds, indicating that the most stable molecular clusters of FmocFF and BPY are held by the hydrogen bonding interactions between the carboxylic acid group of FmocFF and pyridine of BPY (Fig. 5c and Supplementary Table 2). The similar results were obtained for six kinds of trimmers of 2:1 FmocFF/BPY (Fig. 5d and Supplementary Table 2). In addition, considering the steric factors, it was also revealed that O–Ha⋯N hydrogen bond facilitated the formation of long-range ordered molecular organization associated with one-dimensional nanostructures.

### All-atom molecular dynamic simulations

**Fig. 6:**
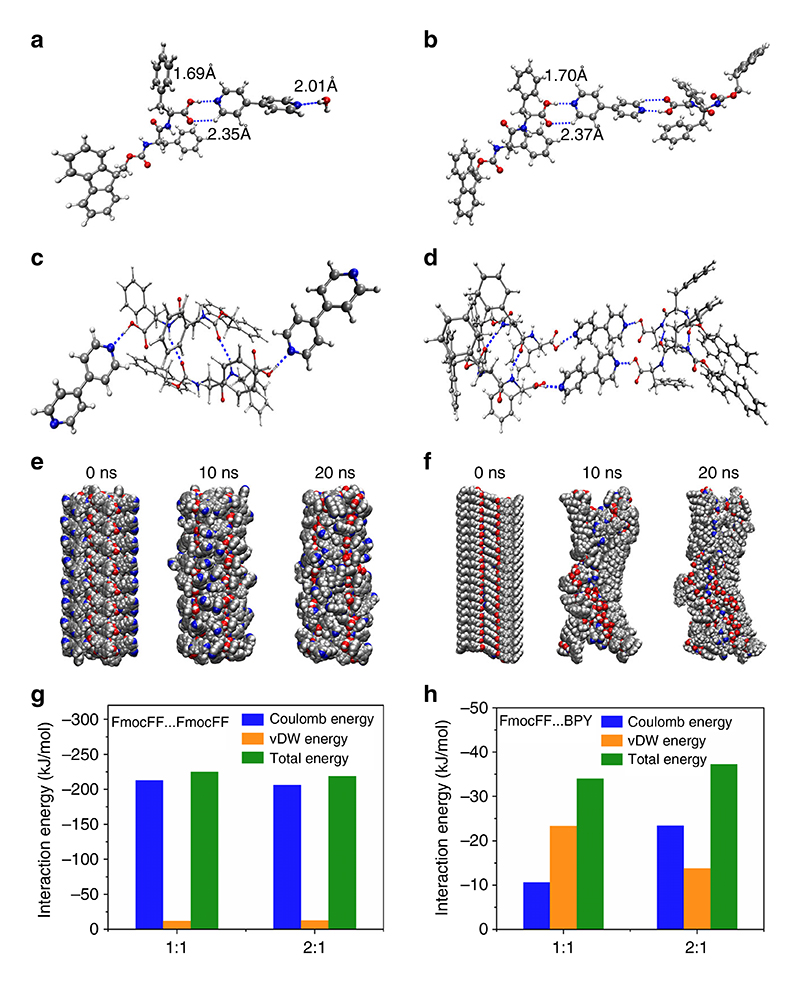
MD simulations for the structural transition of co-assembly. **a**, **b** Molecular clusters of FmocFF and BPY at a ratio of (**a**) 1:1 and (**b**) 2:1. **c**, **d** The elementary building block for β-sheet with co-assembly ratio of (**c**) 1:1 and (**d**) 2:1. **e**, **f** Snapshots of FmocFF-BPY co-assemblies at a ratio of (**e**) 1:1 and (**f**) 2:1 at different intervals obtained from AAMD simulations. **g**, **h** The interaction energies between (**g**) FmocFF peptides and (**h**) FmocFF-BPY

To study the stoichiometry-controlled secondary structure transition of the coassembly systems, computational simulations were performed. Based on the most stable dimer and trimer conformation of FmocFF and BPY (1:1 and 2:1), we further analyzed the effects of the molar ratio of FmocFF to BPY on the intermolecular interaction of these two building blocks (Fig. 6a, b). It was found that when the ratio was 1:1, two types of hydrogen bonds, including O–H⋯N and C–H⋯O’ were formed. In addition, another nitrogen atom in the BPY molecule could interact with water molecules in the solvent. In sharp contrast, when the ratio was changed to 2:1, one BPY molecule would interact with two FmocFF molecules through the formation of four hydrogen bonds, thereby limiting the freedom of the BPY molecule. Combined with the aforementioned spectroscopic and morphology results, the starting molecular packing models were constructed based on the stable conformation of FmocFF homoclusters and FmocFF-BPY heteroclusters at different ratios (1:1 and 2:1) (Fig. 6c, d and Supplementary Fig. 12). The possibility of structural transition was further examined through AAMD simulations. We investigated the structural evolution of the initial molecular models and probed the structural characteristics of the stable final assemblies. Root mean square deviation (RMSD) was calculated relative to the structure present in the initial configuration during the 20 ns of MD simulation. After the first 10 ns, all three structures achieved a stable conformation throughout the simulation. An RMSD change less than or around 2 nm indicates that the biomacromolecule is intact, while larger values mean that it disintegrates^65^. The RMSD graphs (Supplementary Fig. 13) indicated that FmocFF and 1:1 FmocFF/BPY had stable configurations with RMSD values of less than 2 nm, while 2:1 FmocFF/BPY was unstable, with an RMSD value larger than 2 nm. After 20 ns AAMD simulations, it is obvious that the 1:1 co-assembly and the pure FmocFF selfassembly systems almost kept the antiparallel β-sheets conformation, indicating such a secondary structure is energy favorable for both FmocFF assemblies (Fig. 6e and Supplementary Fig. 12). In sharp contrast, the starting model for the 2:1 coassembly quickly became unstable and underwent a structural transition from β-sheet to helix-featured morphology (Fig. 6f). The radius of gyration (Rg) was also calculated during the simulation to analyze the changes in the compactness of the structures (Supplementary Fig. 13). The Rg value maintained an almost constant value for β-sheets conformations, while for the compact-shape helical conformation, the value decreased (from 2.56 to 2.41 nm) over time.

The strong hydrogen bonding interactions between FmocFF and BPY allowed the molecular rearrangement in order to decrease the energy of the overall system and enhance the intermolecular interactions. The mixture of FmocFF/BPY (2:1) could co-assemble to form a stable helical trimer through intermolecular hydrogen bonds and further assembled to generate a supramolecular helical conformation (Supplementary Fig. 14). Furthermore, the interaction energy analysis (Fig. 6g, h and Supplementary Fig. 12) revealed that the Coulomb and overall interaction energies between FmocFF in co-assembly systems decreased compared to those of FmocFF self-assembly systems, indicating the decreasing hydrogen bonding interaction between peptides due to the competition of BPY with FmocFF. In parallel, the Coulomb and overall interaction energies between FmocFF and BPY for the 2:1 co-assembly system is higher than those for the 1:1 system, revealing an elevated hydrogen bonding interaction. In addition, the interaction energies between FmocFF and water molecules demonstrated that water-mediated hydrogen bonding interaction also contributed to the stability of β-sheet structures. Notably, compared to the co-assembly system, the assemblies formed by individual FmocFF possessed the highest overall interaction energies between the peptides, indicating that the introduction of BPY significantly decreased the interaction between peptides. The atomic distance variation of the hydrogen bonds was also calculated during the 20 ns of MD simulation. The result showed that the average atomic distances of the H-bonds decreased from 2.96 to 2.92 and 2.73 nm, while increased average H-bonds distances were observed by introducing BPY in the coassembly states (Supplementary Fig. 15). Taken together, we can conclude that the competition of hydrogen bonding interactions between FmocFF and BPY determines the structure conformation of the co-assemblies.

Aromatic π-π interactions also play a major role in stabilizing the three-dimensional structural conformation in the supramo-lecular gels, and were studied by CD spectra, fluorescence emission spectra, and MD simulations. Aromatic interactions between fluorenyl groups were formed in the gels, accounting for the positive peak at 302 nm and the negative peak at 308 nm in the CD spectra (Fig. 3a–c). Compare with the maximum emission peaks of FmocFF and FmocFF/BPY (2:1, 1:1) in DMSO solution, red shift was observed for all the hydrogels (from around 314 nm to 325, 393, and 411 nm, respectively), indicating π-π interactions of Fmoc rings in the self- and co-assembled states (Supplementary Fig. 16). The increased red shift and broader emission peaks can be attributed to the stabilization of excitons in the coassembled supramolecular hydrogels^66^. Moreover, MD simulations were performed to analyze the π interactions of the self- and co-assembly systems, and the results are showed in Supplementary Figs. 17–20. For FmocFF self-assembly (Supplementary Fig. 18), aromatic π stackings were found for Fmoc⋯ Fmoc and Fmoc⋯ obenzene with centroid distances of 3.45 and 3.23 Å, respectively. T-shape C–H⋯π interactions were also observed by the C1–H1⋯Fmoc (2.75 Å), C32-H32⋯Fmoc (2.89 Å), and C19-H19⋯benzene (2.85 Å). For 1:1 FmocFF/BPY co-assembly (Supplementary Fig. 19), aromatic π stackings of Fmoc⋯Fmoc (3.52 Å), Fmoc⋯benzene (3.61 Å), Fmoc⋯pyridine (3.55 Å), and benzene⋯pyridine (3.51 Å) were observed, suggesting more types of aromatic π interactions in the co-assembly state. C–H⋯π interaction was also found for the C29-H29⋯Fmoc (2.61 Å). There were two types of Fmoc⋯Fmoc interactions (3.48 and 3.43 Å), and benzene⋯pyridine (3.54 Å) in the 2:1 FmocFF/BPY co-assembly with a T-shape stacking of C38-H38⋯ benzenebenzene (2.51 Å) (Supplementary Fig. 20).

## Discussion

In summary, the stoichiometry-controlled secondary structure transition from amyloid-like β-sheet to helix has been successfully achieved by co-assembly of the FmocFF dipeptide and achiral bipyridine derivative through the competition of hydrogen bonding interactions. The co-assembly triggered transition of secondary structure was fully demonstrated by CD, TEM, FTIR, and molecular dynamics simulations. It exemplifies a feasible strategy to simplify the complexity of proteins and study the inhibition of short dipeptide building blocks amyloid-like structures formation. Moreover, the biocompatibility of the FmocFF/BPY co-assembly was demonstrated via an MTT-based cell viability assay for both the human HeLa cervical cancer and SH-SY5Y human neuroblastoma cell line (Supplementary Fig. 21). The present work paves a new way to explore the transition of secondary structures through co-assembly and may lay the basis for various applications, including neurodegenerative diseases therapeutics, cell culture and tissue engineering.

## Methods

### Materials

All the solvents and chemicals are commercially available. Chemicals were used without further purification. Water was processed using a Millipore purification system (Darmstadt, Germany) with a minimum resistivity of 18.2 MΩ cm. 4,4-BPY was purchased from Sigma, and ultrapure water was obtained from Biological Industries. Fmoc-modified diphenylalanine (FmocFF) was purchased from Bachem at a purity level of >98%.

### Chemical identity for FmocFF and BPY

^1^H and ^13^C NMR spectra were recorded in deuterated solvent on a Bruker Advance 400 MHz spectrometer. The ^1^H NMR chemical shifts (δ) are given in ppm referring to internal standard tetramethylsilane (TMS). All coupling constants (*J*) are given in Hz. FmocFF: ^1^H NMR (400 MHz, DMSO-d_6_) δ: 12.67 (s, 1H), 8.20 (d, *J* = 8.0 Hz, 1H), 7.80 (d, *J* = 4.0 Hz, 2H), 7.48–7.57 (m, 3H), 7.31–7.34 (m, 2H), 7.08–7.21 (m, 12H), 4.38–4.43 (m, 1H), 4.17–4.23 (m, 1H), 4.01–4.08 (m, 3H), 2.99–3.03 (dd, *J_1_* = 4.0 Hz, *J_1_* = 12.0 Hz, 1H), 2.85–2.92 (m, 1H), 2.60–2.69 (m, 1H). ^13^C NMR (100 MHz, DMSO-d_6_) δ: 173.0, 171.9,156.0,144.1,144.0, 140.9, 138.4, 137.6,129.5,129.4,128.5,128.3,127.9,127.3, 126.7, 126.5, 125.7, 125.6, 120.4, 66.7, 65.9, 56.28, 53.79, 46.88, 37.3, 37.0, 23.1. MS (ESI) calcd for C33H30N2O5 [M+H]^+^, 535.2; found, 535.6. BPY: ^1^H NMR (400 MHz, DMSO-dg) δ: 8.72–8.74 (dd, *J_1_* = 4.0 Hz, *J_1_* = 8.0 Hz, 4H), 7.83–7.85 (dd, *J_1_* = 4.0 Hz, *J_1_* = 8.0 Hz, 4H). ^13^C NMR (100 MHz, DMSO-d_6_) δ: 150.9, 144.6, 121.6. MS (ESI) calcd for C10H8N2 [M+H]^+^, 157.0; found, 157.3. ^1^H NMR, ^13^C NMR and MS spectra of FmocFF and BPY are shown in Supplementary Figs. 22–27.

### Gel preparation

Ten microliters dimethyl sulfoxide (DMSO) solution of FmocFF or mixture of FmocFF/BPY was added to 490 μL water to a total gelator concentration of 2.0 mg/mL. Gel formation was evaluated by the “invert-vial” method.

### Turbidity assay

One hundred microliters solutions of FmocFF, BPY, 2:1 mol ratio of FmocFF/BPY and 1:1 mol ratio of FmocFF/BPY were made as described above and inserted into a 96-well plate. Absorbance at 405 nm was measured every 10 s using a TECAN Infinite M200PRO plate reader, for a total time of 100 min.

### CD spectroscopy

CD spectra of gels were collected on a JASCO J-820 CD Spectrometer with bandwidth of 1.0 nm in the ultraviolet (UV) region (190–400 nm) using a 0.1 mm quartz cuvette. All scans were performed at a scan speed of 200 nm min^−1^ with a data pitch of 0.5 nm at room temperature. All spectra were obtained following solvent background subtraction. The reported spectra were the average of 3 scans.

### TEM images

Aliquots (10 μL) of sample solution were added into a glow discharge copper grid (400 mesh) coated with thin carbon film. Excess solution was then removed, the grid was washed with deionized water three times and stained with 2.0% (w/v) uranyl acetate (UA) by exposing the grid to one drop of UA solution for 10 s. TEM images were viewed using a JEOL 1200EX electron microscope operating at 80 kV.

### FTIR spectroscopy

Seven hundred and fifty microliters of gel was deposited onto a real crystal KBr IR card (International Crystal Labs, Garfield, New Jersey, USA) and vacuum dried. The dried sample was then wetted three times with 1 mL of D_2_O. The FTIR spectra were recorded on a Nicolet 6700 FTIR spectrometer (Thermo Scientific, Waltham, Massachusetts, USA), from 1700 to 1600 cm^−1^ at room temperature. One twenty eight scans were collected with a spectral resolution of 4 cm^−1^ in nitrogen atmosphere. The background signal was recorded using D2O and subtracted to obtain each FTIR spectrum. The IR spectra of the amide I region (1600–1700 cm^−1^) were then fitted by multiple Gaussian peaks, and the estimated proportion of each secondary structure constituent was calculated using the OriginPro software.

### Rheology

Rheological studies were performed on an ARES-G2 rheometer (TA Instruments, New Castle, DE, USA) using a 20 mm parallel-plate geometry with a gap of 1000 μm. For time sweep experiments, the gels were prepared on the plate the time sweep was carried out at constant frequency of 1 Hz and 0.1% strain. Otherwise, 1-day aged gels were used. Strain sweep was carried out at a constant frequency of 1 Hz. Dynamic frequency sweep experiments were carried out at a constant strain of 0.1%. Sheer recovery was probed by the step strain method, with time sweep steps carried out at 0.1 and 200% strain successively.

### NMR spectrometry for hydrogen bonding study

Powders of FmocFF and BPY at different ratios were dissolved in dimethyl sulfoxide-*d*_6_ (DMSO-*d*_6_) at a final FmocFF concentration of 4.0 mg/mL. The ^1^H NMR spectra were recorded on a Bruker Advance 400 MHz spectrometer. For temperature-dependent experiment, 250 μL DMSO-*d*_6_ solution of mixture of FmocFF/BPY (1:1) was added to 250 μL D2O in a total gelator concentration of 1.0 mg/mL for overnight incubation. A 500-MHz ^1^H NMR spectrometer (DMX500 spectrometer, Bruker) was used to detect spectral changes at various temperatures. The temperature was equilibrated for 10 min before the measurements.

### Computational methods

AAMD simulations are performed using Gromacs package (Version 5.1.4)^67^. The general AMBER force field (GAFF) was used to model the Fmoc-FF and BPY molecules. The GAFF potential is well developed and it was previously used to compute processes of aggregation and crystal growth of organic molecules. Water molecules were modelled using the tip3p potential. To derive the force field parameters within the framework of the GAFF, the geometry optimization and molecular electrostatic potential of Fmoc-FF and BPY were obtained at the level of HF/6-31 g(d) theory. The Antechamber package was then used to compute partial charge according to the restrained electrostatic potential (RESP) formalism. Moreover, the basic molecular packing units corresponding to different experimental conditions were optimized at the level of B3LYP/6-31 g(d,p) theory. The harmonic vibrational frequency calculations on the optimized geometries were also performed to ensure the structures at local minima. All the quantum chemical calculations were performed in the Gaussian 09 package^68^. The hydrogen bond strength was determined through the quantum theory of atoms in molecules (QTAIM) analyzes using the Multiwfn program^69^. The FmocFF-BPY binding energies (BE) were calculated as the energy difference between their molecular clusters and the sum of the energies of FmocFF and BPY (Eq. 1).

(1) BE=E(mFmocFF−nBPY)−mE(FmocFF)−nE(BPY)

AAMD simulation was performed on water boxes with different ratios of FmocFF to BPY, where the number of FmocFF was fixed at 48. The solution was at first minimized using the conjugate-gradient algorithm with a tolerance on the maximum force of 200 KJ/mol, and the temperature and volume of each system were equilibrated by running 400 ps of constant volume, constant temperature (NVT) simulation, followed by 400 ps NPT simulations. Production runs in the NPT ensemble were then conducted for 20 ns. The leapfrog algorithm with a time step of 2 fs was used to integrate the equations of motion. The isothermal-isobaric (constant NPT) ensemble was used to maintain a temperature of 300 K and a pressure of 1 bar. The velocity rescale thermostat and the isotropic Parrinello-Rahman barostat were used with relaxation times of 0.4 and 2.0 ps, respectively. The electrostatic forces were calculated by means of the particle-mesh Edwald approach with a cutoff of 1.0 nm. A 1.0 nm cutoff was also used for the van der Waals forces. The LINCS algorithm was applied at each step to preserve the bond lengths.

### Cell viability assay

Human cervical cancer cells (HeLa) and human neuroblastoma cells (SH-SY5Y) were cultured in DMEM supplemented with 10% FBS and 1% penicillin/streptomycin at 37 °C in a humidified 5% CO_2_ incubator. For cell viability studies, 2 × 10^5^ cells/ ml were cultured in 96-well tissue microplates (100 μl per well) and were allowed to adhere overnight at 37 °C. The medium was then replaced with a medium containing FmocFF/BPY co-assembly at different BPY concentrations (10–100 μM in cell culture medium). Cells were incubated for 24 h and 10 μL 3-(4,5-dimethylthiazolyl-2)-2,5-diphenyltetrazolium bromide (MTT) stock solution (5 mg/mL) in phosphate buffer saline was added to each well and incubated for 4 h at 37 °C. Next, 100 μl of extraction buffer [20% SDS dissolved in a solution of 50% N,N-dimethylformamide and 50% double-distilled water (pH 4.7)] were added to each well, followed by incubation at 37 °C for 30 min. The resulting absorbance at 570 nm was measured using a microplate reader. The results presented are the mean of three independent experiments ± the standard error of the mean.

## Supplementary Material

Supplementary material

## Figures and Tables

**Fig. 1:**
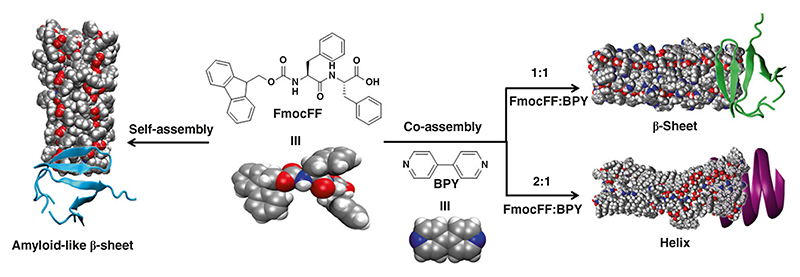
Co-assembly induced structural transition. Schematic presentation of stoichiometry-controlled secondary structural transition of amyloid-derived dipeptide assemblies (FmocFF) from a β-sheet to a helix structure through co-assembly with 4,4-bipyridine (BPY)

## Data Availability

The data supporting the findings of this study are available within the article and its Supplementary Information file or directly from the corresponding author upon reasonable request.

## Terms and Conditions

Springer Nature journal content, brought to you courtesy of Springer Nature Customer Service Center GmbH (“Springer Nature”).

Springer Nature supports a reasonable amount of sharing of research papers by authors, subscribers and authorised users (“Users”), for smallscale personal, non-commercial use provided that all copyright, trade and service marks and other proprietary notices are maintained. By accessing, sharing, receiving or otherwise using the Springer Nature journal content you agree to these terms of use (“Terms”). For these purposes, Springer Nature considers academic use (by researchers and students) to be non-commercial.

These Terms are supplementary and will apply in addition to any applicable website terms and conditions, a relevant site licence or a personal subscription. These Terms will prevail over any conflict or ambiguity with regards to the relevant terms, a site licence or a personal subscription (to the extent of the conflict or ambiguity only). For Creative Commons-licensed articles, the terms of the Creative Commons license used will apply.

We collect and use personal data to provide access to the Springer Nature journal content. We may also use these personal data internally within ResearchGate and Springer Nature and as agreed share it, in an anonymised way, for purposes of tracking, analysis and reporting. We will not otherwise disclose your personal data outside the ResearchGate or the Springer Nature group of companies unless we have your permission as detailed in the Privacy Policy.

While Users may use the Springer Nature journal content for small scale, personal non-commercial use, it is important to note that Users may not:

1. use such content for the purpose of providing other users with access on a regular or large scale basis or as a means to circumvent access control;
2. use such content where to do so would be considered a criminal or statutory offence in any jurisdiction, or gives rise to civil liability, or is otherwise unlawful;
3. falsely or misleadingly imply or suggest endorsement, approval, sponsorship, or association unless explicitly agreed to by Springer Nature in writing;
4. use bots or other automated methods to access the content or redirect messages
5. override any security feature or exclusionary protocol; or
6. share the content in order to create substitute for Springer Nature products or services or a systematic database of Springer Nature journal content.

In line with the restriction against commercial use, Springer Nature does not permit the creation of a product or service that creates revenue, royalties, rent or income from our content or its inclusion as part of a paid for service or for other commercial gain. Springer Nature journal content cannot be used for inter-library loans and librarians may not upload Springer Nature journal content on a large scale into their, or any other, institutional repository.

These terms of use are reviewed regularly and may be amended at any time. Springer Nature is not obligated to publish any information or content on this website and may remove it or features or functionality at our sole discretion, at any time with or without notice. Springer Nature may revoke this licence to you at any time and remove access to any copies of the Springer Nature journal content which have been saved.

To the fullest extent permitted by law, Springer Nature makes no warranties, representations or guarantees to Users, either express or implied with respect to the Springer nature journal content and all parties disclaim and waive any implied warranties or warranties imposed by law, including merchantability or fitness for any particular purpose.

Please note that these rights do not automatically extend to content, data or other material published by Springer Nature that may be licensed from third parties.

If you would like to use or distribute our Springer Nature journal content to a wider audience or on a regular basis or in any other manner not expressly permitted by these Terms, please contact Springer Nature at

onlineservice@springernature.com
