# Supplementary material for: Stoichiometry-controlled secondary structure transition of amyloid-derived supramolecular dipeptide co-assemblies
Source: Commun Chem. Author manuscript; Available in PMC 2024 Dec 6. (PMC7617032; doi:10.1038/s42004-019-0170-z)
Supplement: Supplementary material [file EMS117030-supplement-Supplementary_material.pdf]

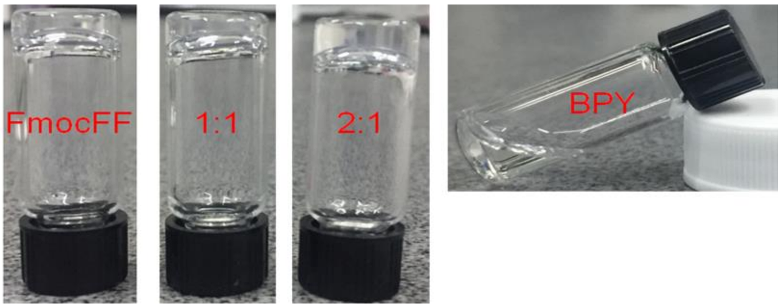

Supplementary Fig. 1 Photos of FmocFF, FmocFF/BPY (1:1 and 2:1) gels and BPY solution prepared at a total gelator concentration at 2.0 mg/mL. Transparent gels were observed for FmocFF and FmocFF/BPY samples, while a clear solution was obtained for single BPY.

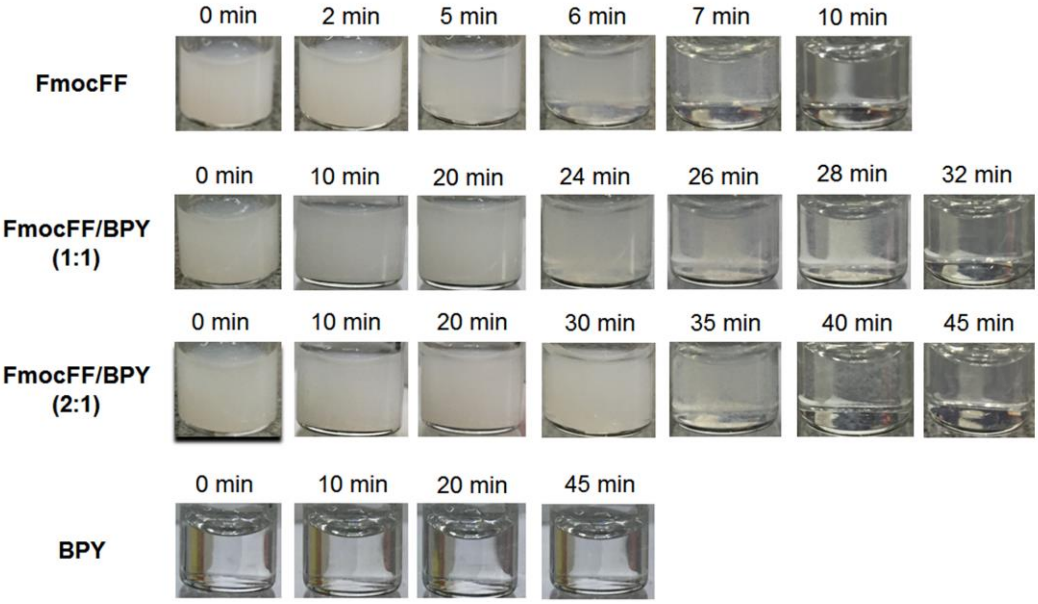

Supplementary Fig. 2 Time lapse images of FmocFF, BPY and FmocFF/BPY at various ratios (1:1, 2:1) in H_2O/DMSO (v/v = 98:2).

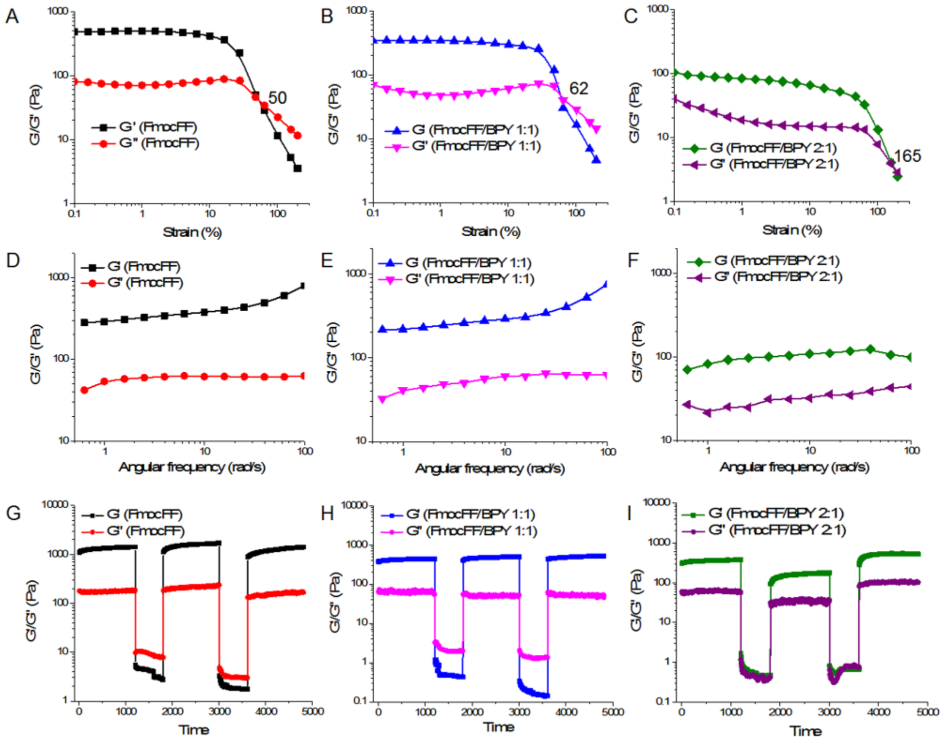

Supplementary Fig. 3 (A-C) Rheological measurement of strain sweep at a constant frequency of 1 Hz. . (D-F) Dynamic frequency sweep at a strain of 0.1% over a range of 0.1-100 rads^{-1}. (G-I) Continuous step strain measurement at alternate 0.1% and 100% strain with time scale. (A, D, G) FmocFF. (B, E, H) 1:1 FmocFF/BPY. (C, F, I) 2:1 FmocFF/BPY.

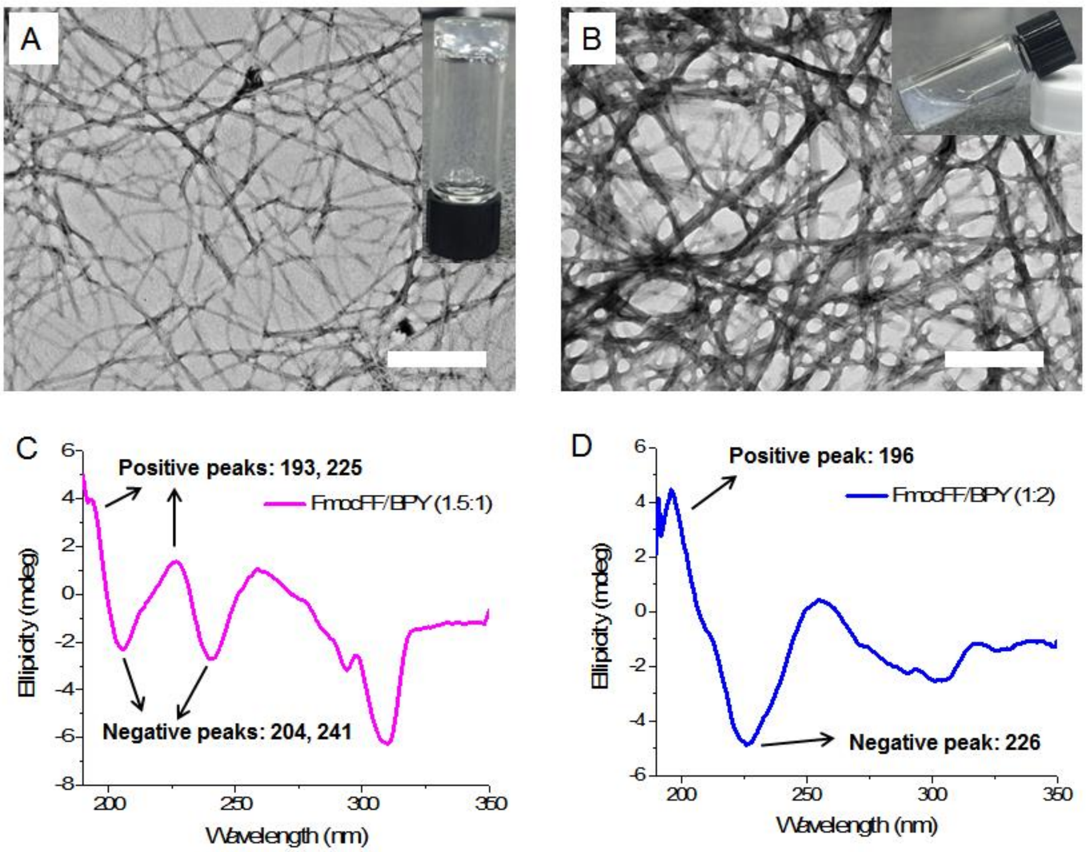

Supplementary Fig. 4 (A-B) TEM images and (C-D) CD spectra of (A, C) 1.5:1 FmocFF/BPY, (B, D) 1:2 FmocFF/BPY in H_2O/DMSO (v/v = 98:2) at a total concentration of 2 mg/mL. The insert in (A-B) is the corresponding optical image.

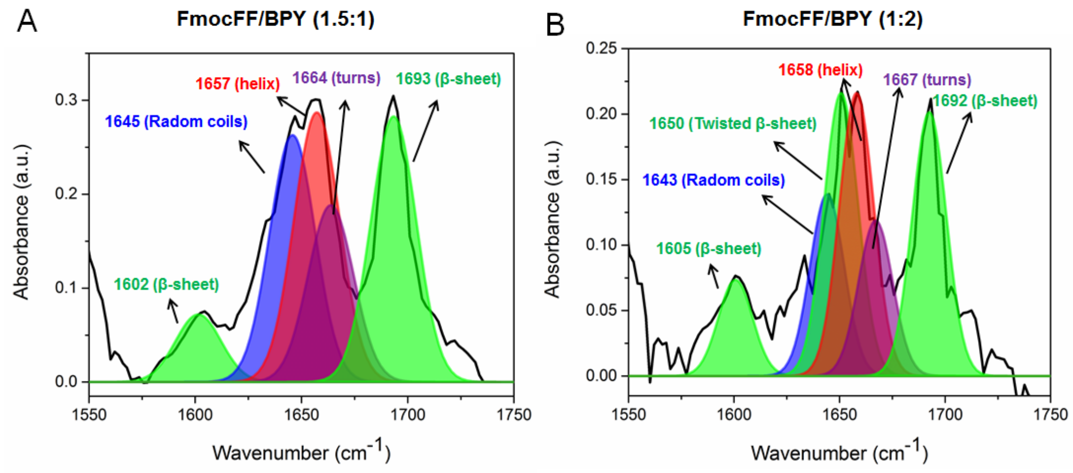

Supplementary Fig. 5 FTIR analysis of the amide I region, ranging from 1,600 to 1,700 cm-1, of FmocFF/BPY mixtures (1.5:1, 1:2) fit by multiple Gaussian peaks.

FmocFF/BPY \ Secondary structures	β-Sheet	Helix	Radom coils	Turns
1:0	1605, 1691<br>(56.63%)	(0%)	1642<br>(33.02%)	1662<br>(10.35%)
2:1	1693<br>(16.04%)	1656<br>(45.71%)	1643<br>(26.14%)	1666<br>(12.11%)
1.5:1	1602, 1693<br>(32.45%)	1657<br>(26.26%)	1645<br>(24.06%)	1664<br>(17.23%)
1:1	1604, 1690,<br>(53.41%)	(0%)	1645<br>(22.20%)	1663<br>(24.38%)
1:2	1602, 1692,<br>1650 [a]<br>(50.89%)	1658<br>(22.40%)	1643<br>(14.38%)	1667<br>(12.31%)
0:1	\<br>(0%)	\<br>(0%)	\<br>(0%)	\<br>(0%)

Supplementary Table 1 Estimated percentage of different secondary structural conformations of FmocFF/BPY gels prepared at different ratios. For 1:0 single FmocFF and 1:1 FmocFF/BPY, the fibril secondary structures mainly consisted of β-sheets (56.63%, 53.41%, respectively). For 1.5:1 and 1:2 FmocFF/BPY, the helix fraction was 26.26% and 22.40% of the total, respectively, while the highest proportion of helix was observed for 2:1 FmocFF/BPY (45.71%). [a] Twisted β-sheet peak (1650 cm^{-1}) in 1:2 FmocFF/BPY.

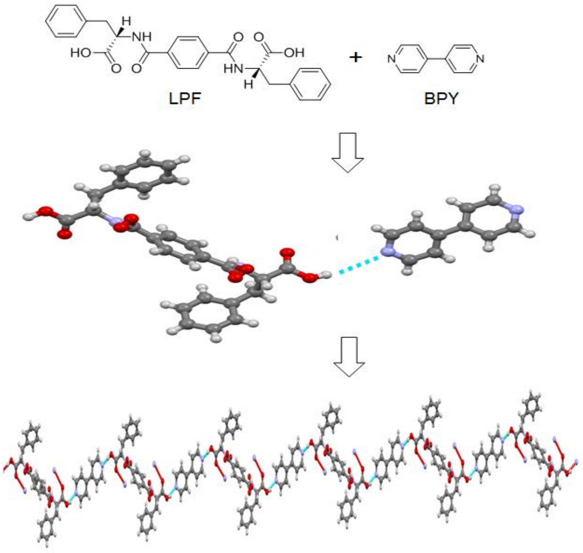

Supplementary Fig. 6 Hydrogen bonding formation between phenylalanine-based derivative (LPF) and BPY in the co-crystal structure.

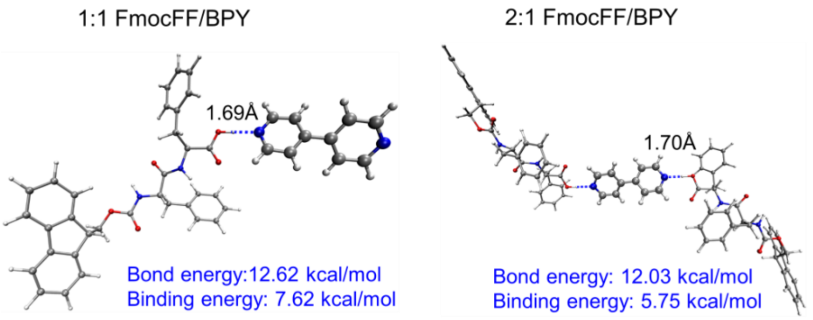

Supplementary Fig. 7 Hydrogen bonding between FmocFF and BPY at a molar ratio of 1:1 and 2:1. The shorter bond length, higher bond strength and binding energy were observed for the stronger hydrogen bond in the dimer of 1:1 FmocFF/BPY.

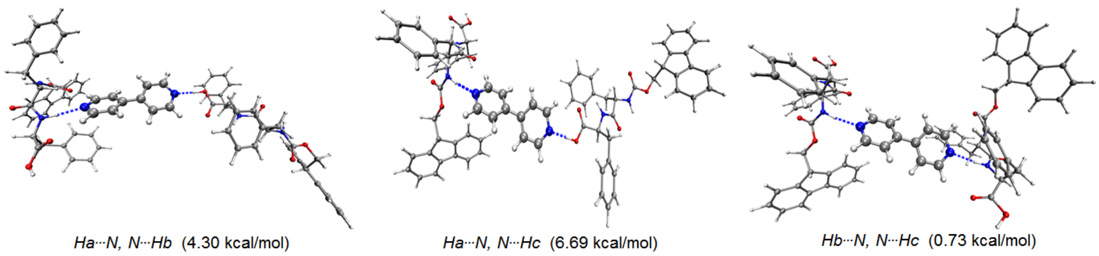

Supplementary Fig. 8 Multiple trimers of FmocFF and BPY connected by different hydrogen bonding interaction modes at a ratio of 2:1 FmocFF/BPY. The corresponding binding energy is shown below the molecular packing patterns.

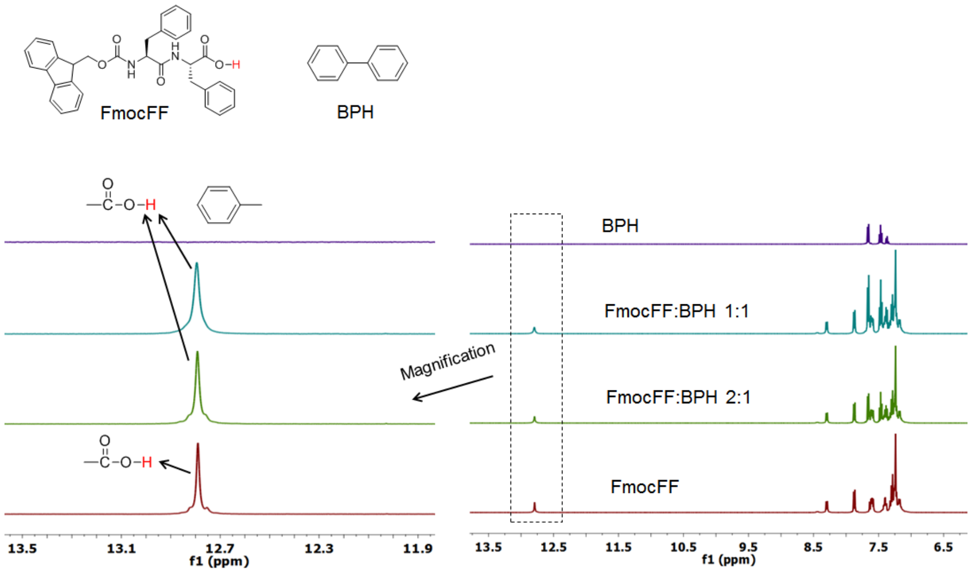

Supplementary Fig. 9 ^{1}H NMR spectra of FmocFF and BPH with different equivalents of BPH in DMSO-d_6. The left part is the proton signal of -COOH corresponding to the right spectra, 10 times magnified. The -COOH peak showed little change by the addition of BPH, indicating no hydrogen bonding formation between -COOH and BPH.

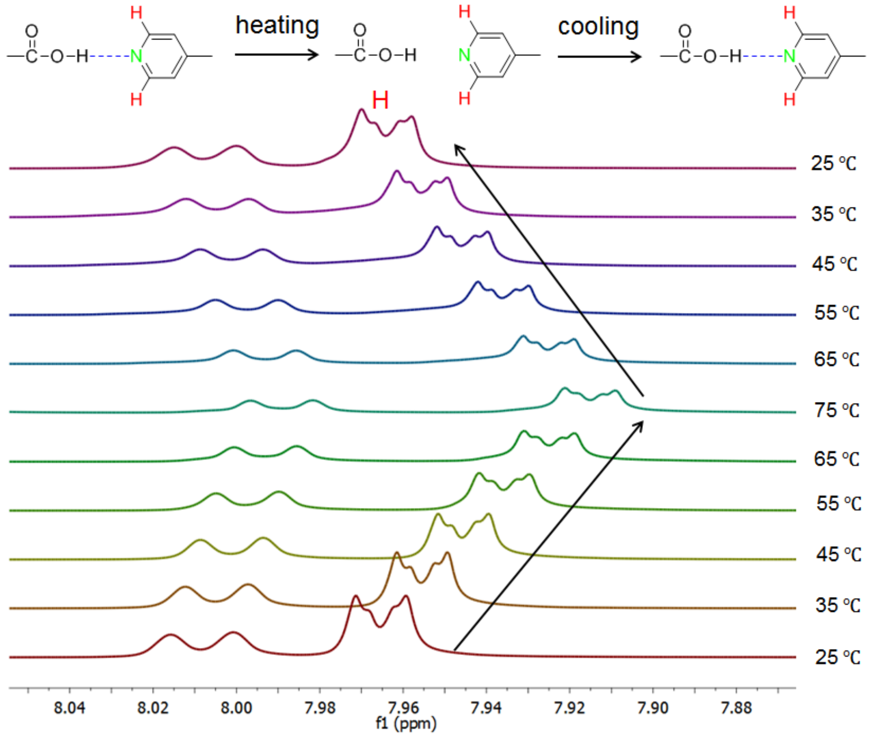

Supplementary Fig. 10 Temperature dependent ^{1}H NMR spectra of 1:1 FmocFF and BPY (1) in DMSO-$d6$/D_2O (1:1) at a total concentration of 1 mg/mL. The upfield

shift of hydrogen (red colored) adjacent to the BPY nitrogen (green colored) was observed due to the disassembly of hydrogen bonding between -COOH and BPY through heating in aqueous solution. The reversible property of this hydrogen bond triggered by temperature was also demonstrated by heating and cooling cycle.

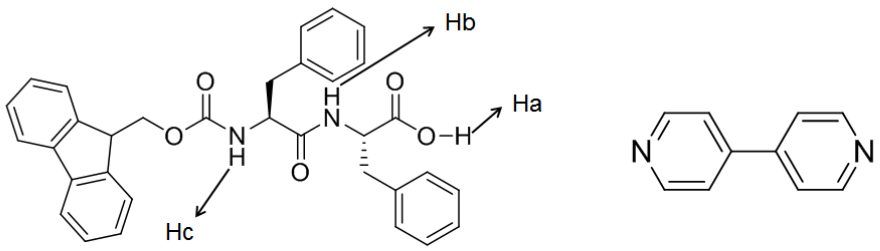

Supplementary Fig. 11 Chemical structures of FmocFF and BPY. Three types of hydrogen atoms (Ha, Hb, Hc) in FmocFF can potentially form hydrogen bond with nitrogen atom in pyridine ring.

	Hydrogen bonds	Bond length (Å)	Bond strength (kcal/mol)	Binding energy (kcal/mol)
Dimers of 1:1 FmocFF/BPY	Ha···N	1.69	12.62	7.62
	Hb···N	2.03	5.27	1.92
	Hc···N	2.03	5.24	1.94
Trimers of 2:1 FmocFF/BPY	Ha···N, N···Ha	1.70, 1.70	12.03, 12.03	11.49
	Hb···N, N···Hb	2.03, 2.03	5.29, 5.29	0.33
	Hc···N, N···Hc	2.03, 2.03	5.29, 5.29	0.35
	Ha···N, N···Hb	1.68, 2.04	12.83, 5.22	4.30
	Ha···N, N···Hc	1.69, 2.05	12.42, 5.06	6.69
	Hb···N, N···Hc	2.03, 2.02	5.26, 5.45	0.73

Supplementary Table 2 Bond length, bond strength of hydrogen bonds and binding energy of dimers and trimers of FmocFF and BPY (1:1, 2:1) with multiple hydrogen bonding interaction modes. The shortest bond length and highest binding energy suggest the most stable hydrogen bond interaction mode for dimers of 1:1

FmocFF/BPY or trimers of 2:1 FmocFF/BPY.

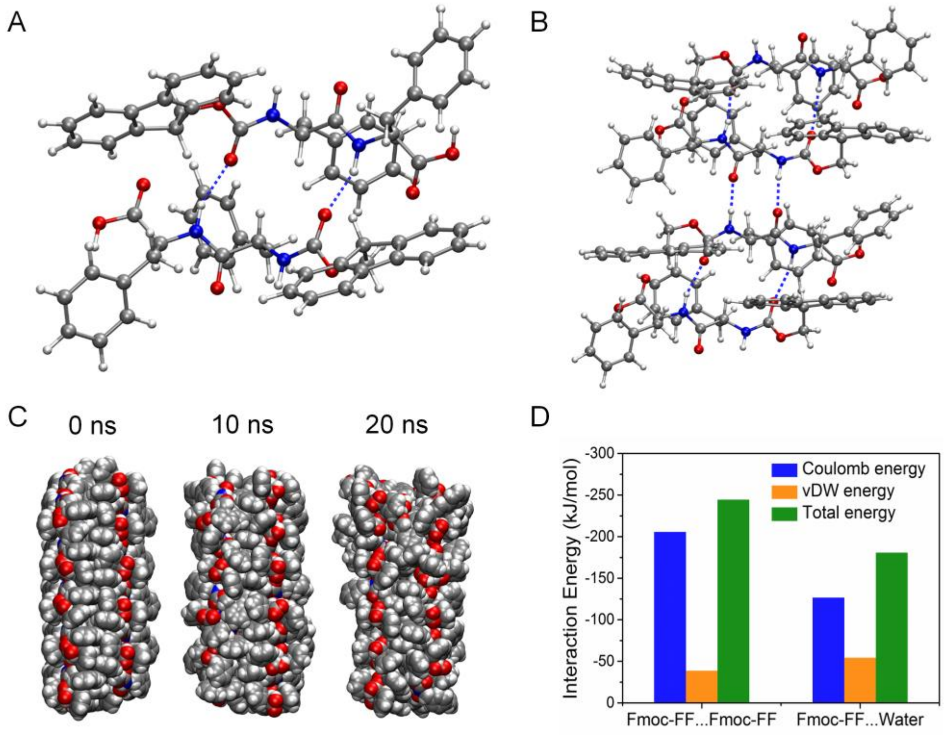

Supplementary Fig. 12 (A) Molecular clusters of FmocFF. (B) The elementary building block for β-sheet structure simulation. (C) Conformation of FmocFF self-assembly at different intervals obtained from AAMD simulations. (D) The interaction energies between FmocFF peptides and between FmocFF and water molecules.

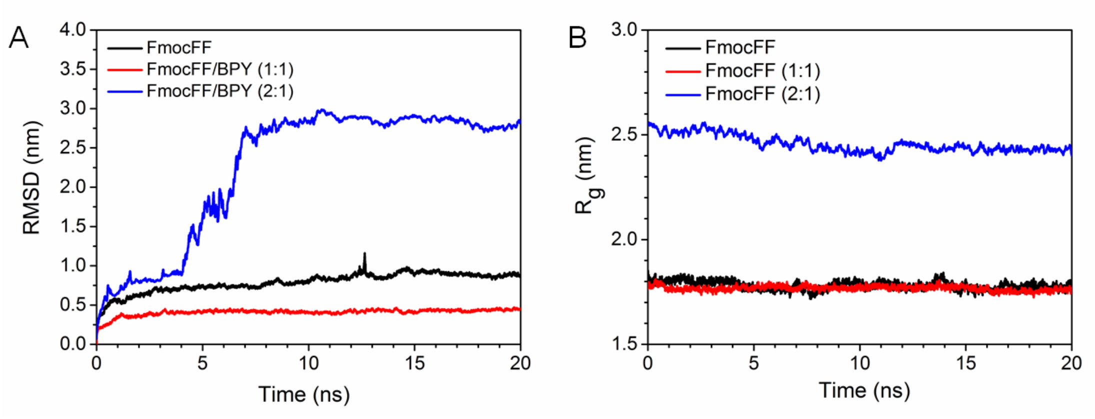

Supplementary Fig. 13 (A) Plots of the RMSD of the backbone atoms of the β-sheet (FmocFF, FmocFF/BPY (1:1)) and helical (FmocFF/BPY (2:1)) conformations versus the starting structures during the 20 ns of MD simulation. (B) Plots of the radius of

gyration (Rg) for FmocFF, FmocFF/BPY (1:1), and FmocFF/BPY (2:1) over the simulation time.

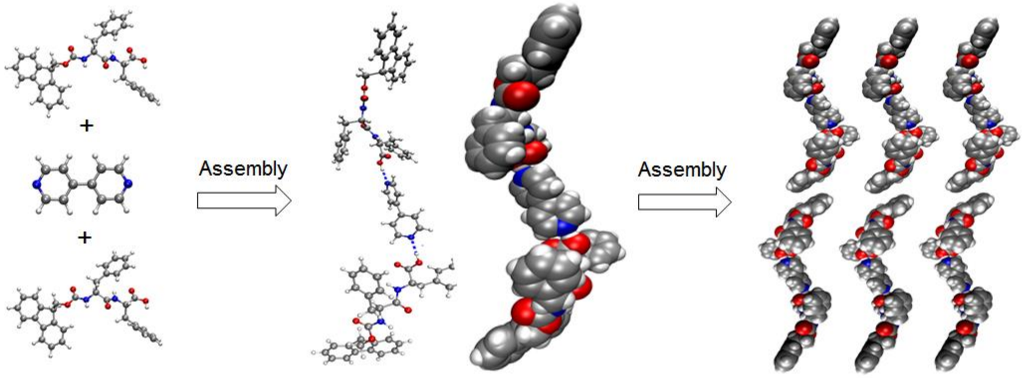

Supplementary Fig. 14 Formation of a hydrogen-bonded helical trimer and further sup ramolecular helical conformation resulting from FmocFF/BPY (2:1) co-assembly. The intermolecular H-bonds are shown as blue dotted lines.

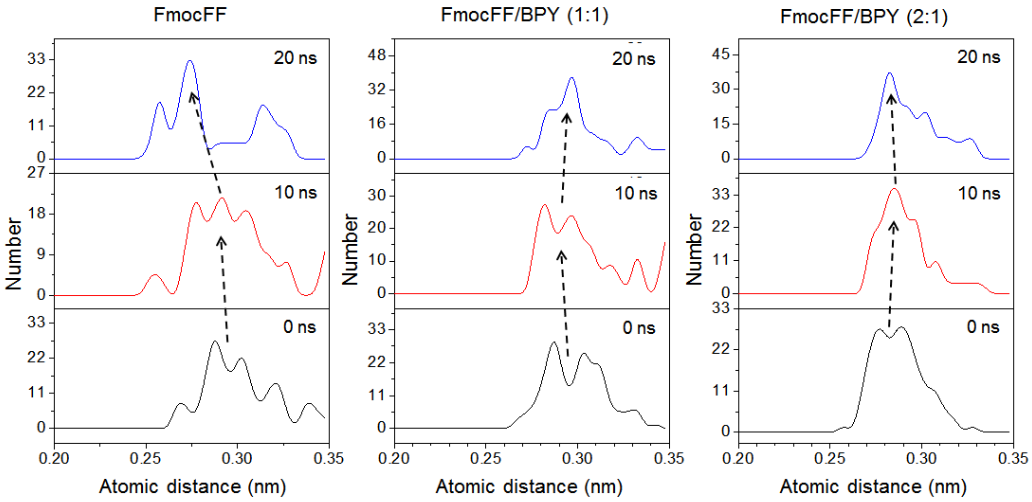

Supplementary Fig. 15 Plots of the atomic distance variation of the H-bonds for FmocFF, FmocFF/BPY (1:1) and FmocFF/BPY (2:1) during the 20 ns of MD simulation.

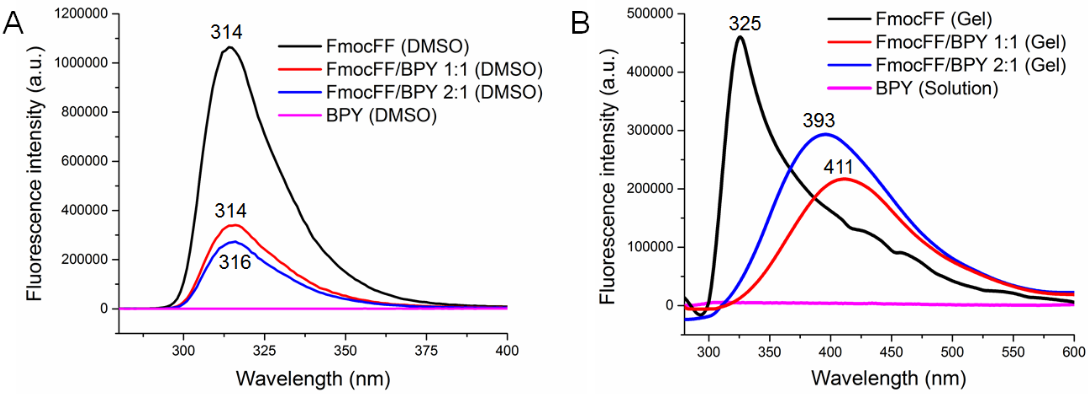

Supplementary Fig. 16 Fluorescence emission spectra of FmocFF, BPY, FmocFF/BPY (2:1, 1:1) in (A) DMSO and (B) H_2O/DMSO (v/v = 98:2) at a concentration of 2 mg/mL.

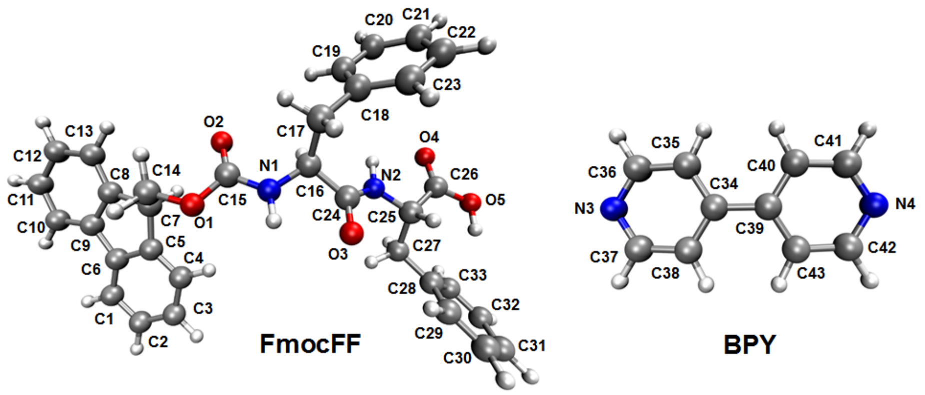

Supplementary Fig. 17 Chemical structures of FmocFF and BPY with different sequence numbers of atoms.

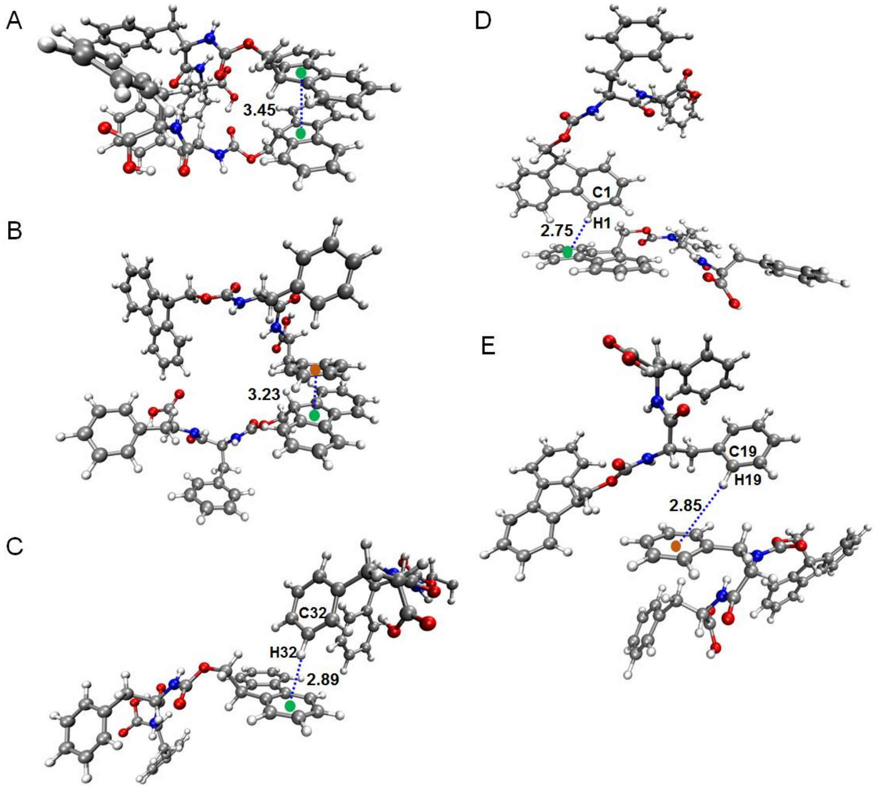

Supplementary Fig. 18 The π stacking interactions of FmocFF self-assembly. (A) Fmoc···Fmoc, (B) Fmoc···benzene, (C) C32-H32···Fmoc, (D) C1-H1···Fmoc, and (E) C19-H19···benzene. The centroids are shown in different colour and the contact distances are shown in blue dashed lines.

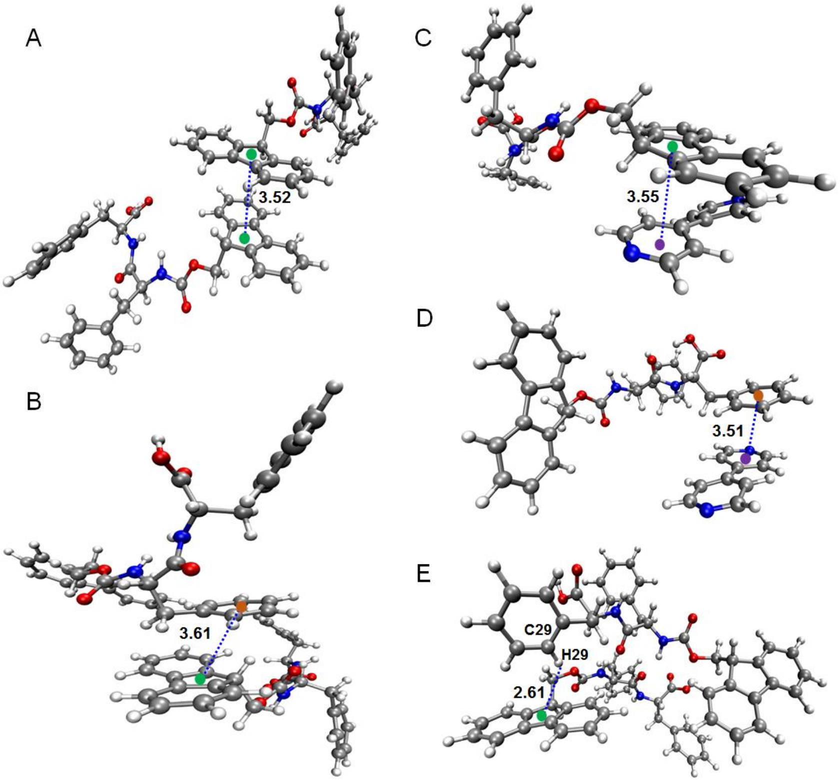

Supplementary Fig. 19 The π stacking interactions of 1:1 FmocFF/BPY co-assembly. (A) Fmoc···Fmoc, (B) Fmoc···benzene, (C) Fmoc···pyridine, (D) Benzene···pyridine, and (E) C29-H29···Fmoc. The centroids are shown in different colour and the contact distances are shown in blue dashed lines.

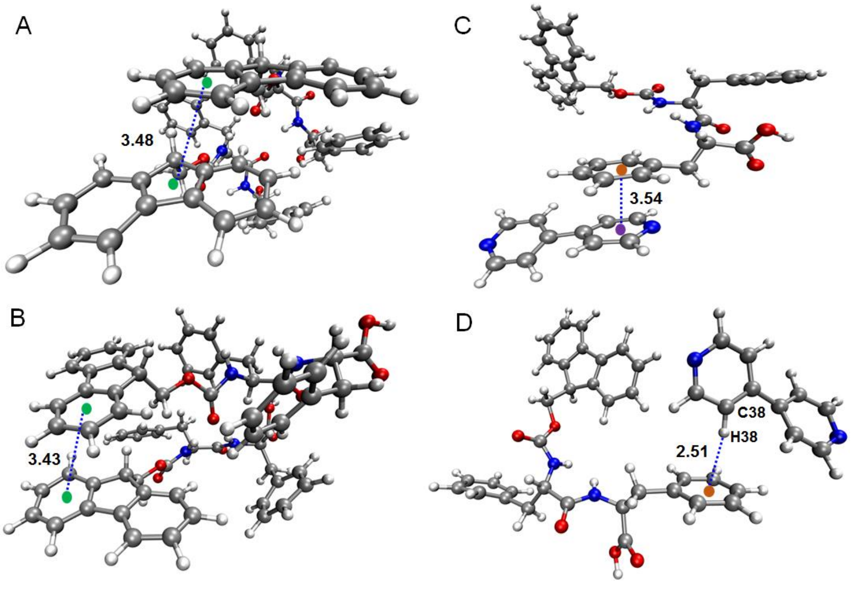

Supplementary Fig. 20 The π stacking interactions of 2:1 FmocFF/BPY co-assembly. (A-B) Fmoc···Fmoc, (C) Benzene···pyridine, and (D) C38-H38···benzene. The centroids are shown in different colour and the contact distances are shown in blue dashed lines.

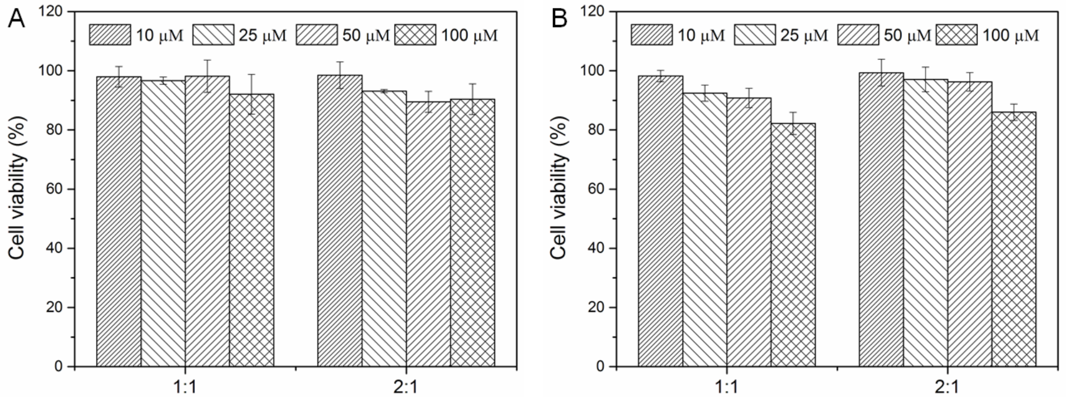

Supplementary Fig. 21 MTT viability assays of (A) human cervical cancer cells (HeLa) and (B) human neuroblastoma cells (SH-SY5Y) incubated for 24 h in media containing different concentrations of BPY in co-assembly of 1:1 FmocFF/BPY and 2:1 FmocFF/BPY. Over 80% of the HeLa and SH-SY5Y cells survived in the presence of BPY in the co-assembly (10-100 μM). Error bars represent standard deviation of the mean.

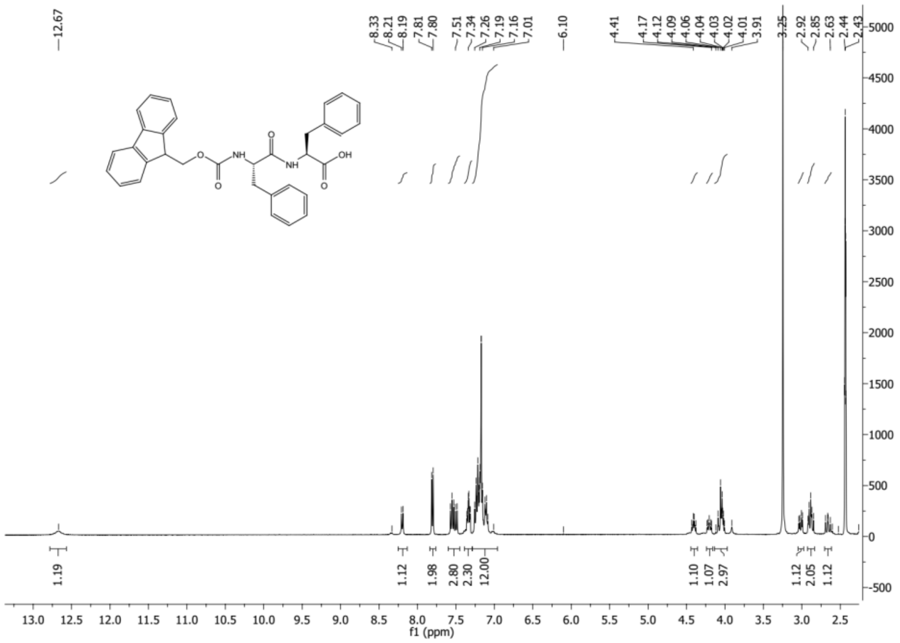

Supplementary Fig. 22 ^{1}H NMR Spectra of FmocFF.

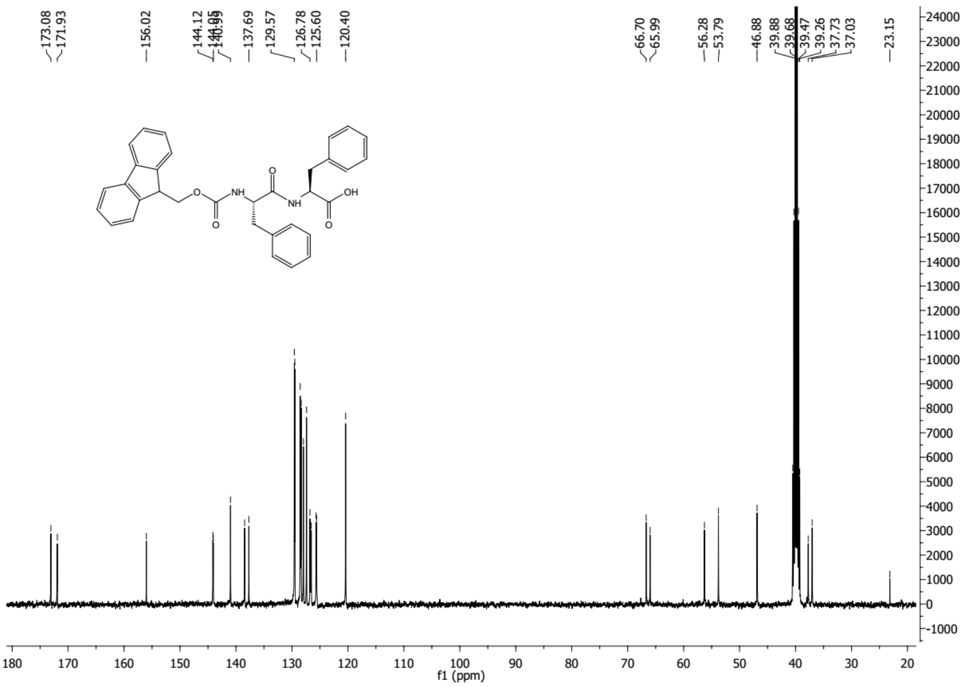

Supplementary Fig. 23 ^{13}C NMR Spectra of FmocFF.

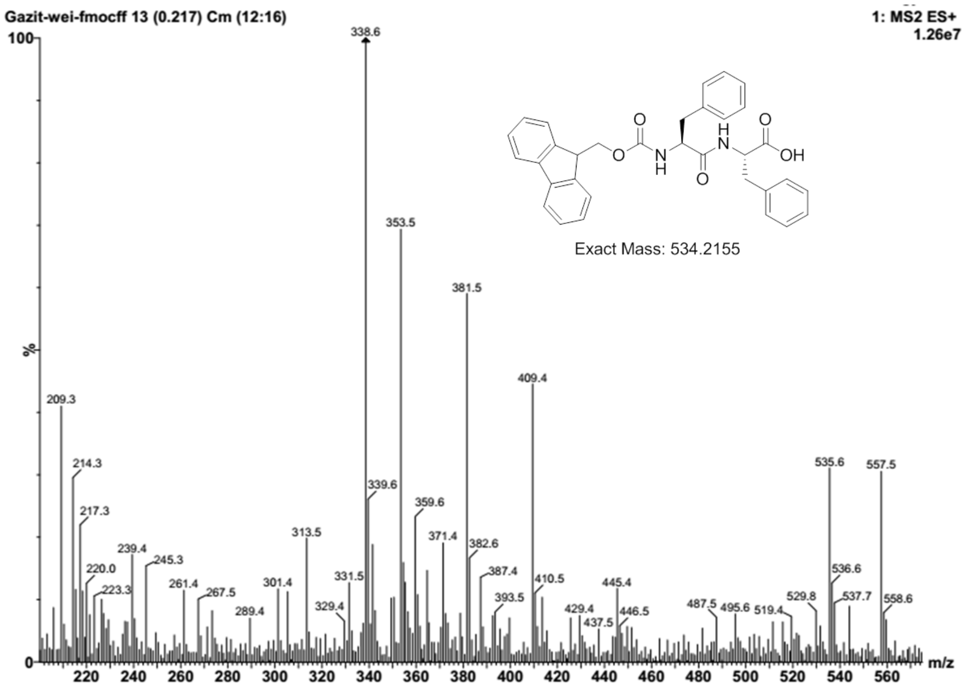

Supplementary Fig. 24 MS Spectra of FmocFF.

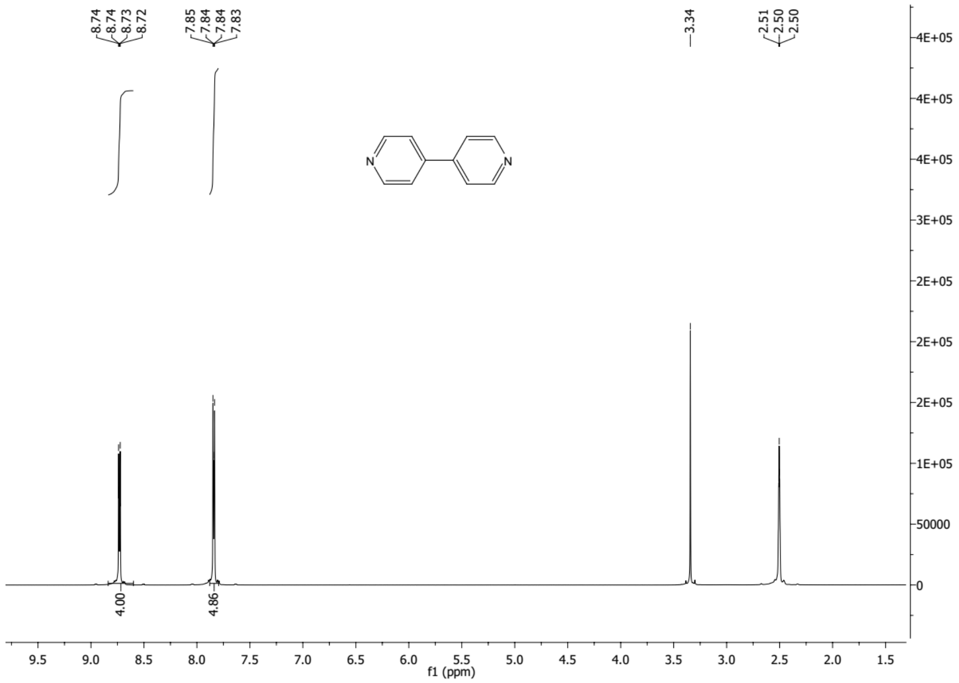

Supplementary Fig. 25 ^{1}H NMR Spectra of BPY.

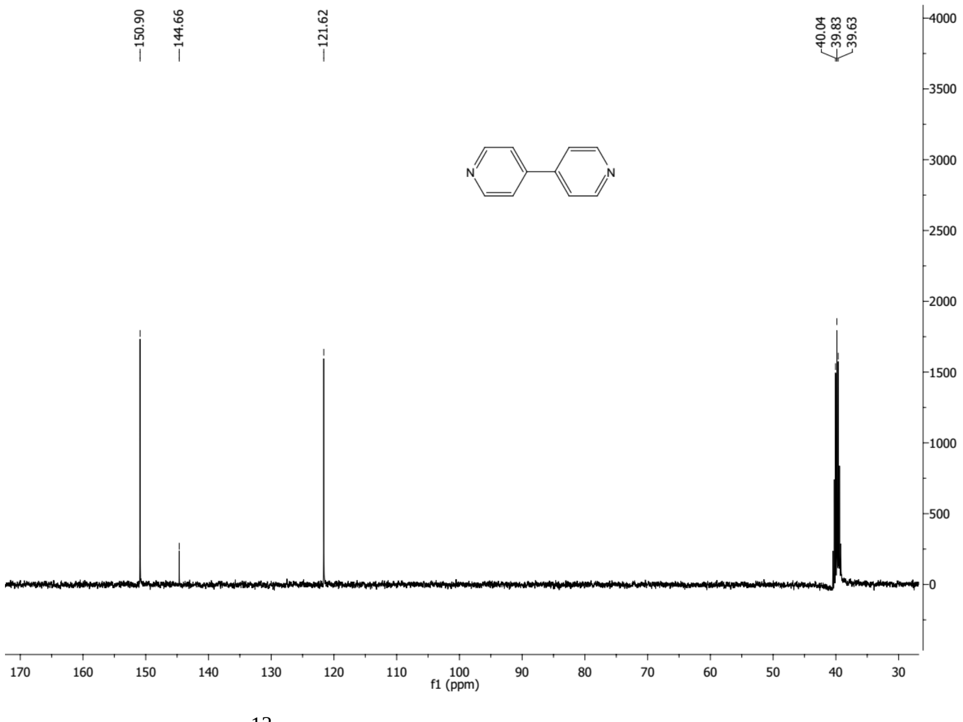

Supplementary Fig. 26 ^{13}C NMR Spectra of BPY.

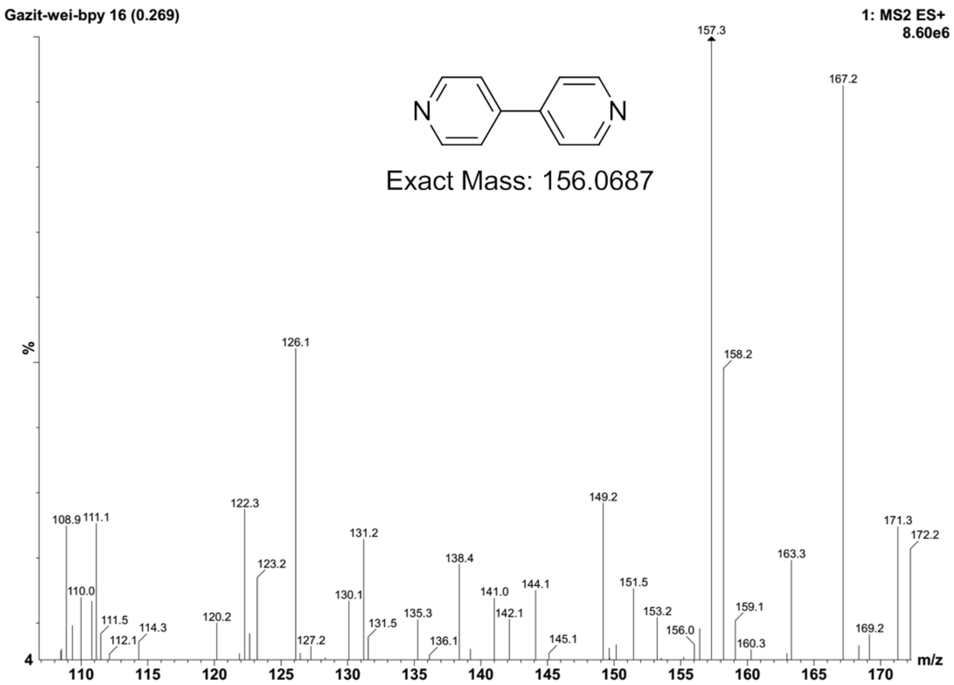

Supplementary Fig. 27 MS Spectra of BPY.
